# Hybrid 2D/3D-quantitative structure–activity relationship studies on the bioactivities and molecular mechanism of antibacterial peptides

**DOI:** 10.1007/s00726-024-03381-x

**Published:** 2024-02-15

**Authors:** Qingguo Yan, Fangfang Wang, Bo Zhou, Xiangna Lin

**Affiliations:** 1 School of Life Science, Linyi University, Linyi, 276000 China; 2 State Key Laboratory of Functions and Applications of Medicinal Plants, College of Basic Medical, Guizhou Medical University, Guizhou, 550004 China

**Keywords:** CoMFA, CoMSIA, Multiple linear regression, Partial least squares regression, Support vector regression

## Abstract

Antimicrobial peptide (AMP) is the polypeptide, which protects the organism avoiding attack from pathogenic bacteria. Studies have shown that there were some antimicrobial peptides with molecular action mechanism involved in crossing the cell membrane without inducing severe membrane collapse, then interacting with cytoplasmic target-nucleic acid, and exerting antibacterial activity by interfacing the transmission of genetic information of pathogenic microorganisms. However, the relationship between the antibacterial activities and peptide structures was still unclear. Therefore, in the present work, a series of AMPs with a sequence of 20 amino acids was extracted from DBAASP database, then, quantitative structure–activity relationship (QSAR) methods were conducted on these peptides. In addition, novel antimicrobial peptides with  stronger antimicrobial activities were designed according to the information originated from the constructed models. Hence, the outcome of this study would lay a solid foundation for the in-silico design and exploration of novel antibacterial peptides with improved activity activities.

**Supplementary Information:**

The online version contains supplementary material available at 10.1007/s00726-024-03381-x.

## Introduction

The emergence of antibiotics is one of the great achievements in the field of medicine in the twentieth century, which are hailed as the “panacea” in medicine (Nikaido 1994; Richmond et al. 2019; Veselinović et al. 2018). Therefore, antibiotics have been widely employed to prevent and treat related diseases caused by microbial infections. However, problems such as multi-drug resistance caused by irrational use of antibiotics and death caused by drug-resistant bacterial infection have become a world problem. Therefore, it is urgent to find novel antibiotics against multi-drug resistant bacteria.

In recent years, scientists have continued to explore novel antibiotics. On one hand, the structures of some antibiotics have been modified to derive more efficient antibiotics. On the other hand, new antibiotics have been screened to break the serious situation faced by traditional antibiotics (Fischbach and Walsh 2009). In 1972, the Swedish scientist Boman discovered the antimicrobial peptide (AMP) for the first time when studying the inducible defense system of the giant silkworm *Hyalophora cecropia* diapause pupae (Boman et al. 1972). As a completely new type of antibiotic, it is expected to break through the barriers of the original antibiotics (Ilyas et al. 2019; Kuhlmann et al. 2018; Shi et al. 2018; Yu et al. 2018; Zanjani et al. 2018). Studies have found that AMPs are encoded by specific gene, which are an important part of the biological natural immune defense system. AMPs have the functions of resisting external microorganisms and removing mutant cells. Therefore, they can not only act on bacteria and fungi, but also have effects on viruses and cancer cells, therefore, AMPs can be treated as conventional antibiotic or coordinated with antibiotics (Zasloff 2002). Furthermore, studies have shown that AMPs have obvious advantages over antibiotics. For example, AMPs would basically get rid of drug dependence on antibiotics due to multi-targets and low probability of drug resistance, additionally, AMPs can specifically act on pathogenic microorganisms and external invasion without toxic or low-toxic to the host. Therefore, AMPs are expected to serve as new, safe and efficient antimicrobial substances (Chung and Khanum 2017; Hanson and Lemaitre 2020; Lee et al. 2018).

The discovered AMPs are basically amphiphilic cationic which would easily attack the cell membrane of pathogenic bacteria, penetrate the cell membrane and cause cell death (Catte et al. 2018; Howl et al. 2018; Silva and Vale 2019). When AMPs interact with pathogenic bacteria, the cell membrane of pathogenic microorganisms is initially contacted, therefore, researches on the mechanism of AMPs are mainly focused on the cell membrane (Catte et al. 2018; Howl et al. 2018; Silva and Vale 2019). However, recent studies have shown that some AMPs cannot damage the cell membrane of bacteria but take macromolecular DNA as the target and further kill the bacteria by affecting gene transcription, expression, and regulation (Park et al. 1998; Rončević et al. 2019).

Assuming that AMPs can interact with the DNA of pathogenic microorganisms, the expression of virulence genes, drug resistance genes or other key genes related to life activities would be inhibited or shut down by affecting or blocking the transmission of genetic information of the pathogenic microorganisms and the synthesis of biological macromolecules, eventually leading to antibacterial effects. However, domestic researches are mainly located on binding phenomena and modes for AMPs-DNA interactions, there are few studies focused on the relationship between the structure of AMPs and the antibacterial activities.

Additionally, studies have proven that penetrating peptides and AMPs are similar in many structural features, for example, they are all cationic peptides with relatively small molecular weights, both form amphiphilic structures when interacting with target membranes (bacteria, fungi, viruses, etc.). In addition, the penetrating peptides also have antibacterial and fungal  activities and are often used as diagnostic or therapeutic carriers, and can even be employed as templates for the development of new penetrating peptides (Nekhotiaeva et al. 2004; Palm et al. 2006; Park et al. 2009; Zhu and Shin 2009). Furthermore, related studies have shown that the penetrating peptide ppTG20 exhibited antibacterial activity, which is mainly derived from higher proportion of hydrophobic amino acids. Similarly, hydrophobicity is also an essential feature for AMP-membrane interactions (Li et al. 2012). It can be seen that the structure and function of penetrating peptide ppTG20 is very similar to AMPs. Therefore, we speculate whether DNA-targeted AMPs can be designed and developed based on the sequence of peptide ppTG20. Thus, the two-dimensional quantitative structure–activity relationship (2D-QSAR) and three-dimensional quantitative structure–activity relationship (3D-QSAR) models were developed on several AMPs with 20 amino acids, which would be useful for studying the mechanism of AMPs and designing of potent peptides with potential use in diseases.

## Materials and methods

### Data sets and biological activity

In the current work, two sets of antibacterial peptides (with sequences of 20 amino acids) targeted on *E. coli* and *Staphylococcus aureus* were chosen from DBAASP database (https://dbaasp.org/home) (Pirtskhalava et al. 2021). All activities were expressed as IC_50_, which were first converted into pIC_50_ (–logIC_50_) values and then employed as dependent variables. In total, the whole dataset was divided into a training set (23 peptides for *Escherichia coli;* 30 peptides for *Staphylococcus aureus*) to construct the QSAR models and a test set (8 peptides for *Escherichia coli;* 10 peptides for *Staphylococcus aureus*) to validate the developed models. The training and test sets are chosen according to the rule that the both sets possess uniform distribution and contain structurally diverse peptides with high, medium and low activity. The sequences and activity data for the taken peptides are shown in Tables 1 and 2.

**Table 1:** Representative skeletons and molecular structures of antibacterial peptides targeted on *Escherichia coli* and the inhibitory affinity pIC_50_ values

Compound	Sequence	pIC_50_(μM)
1	GLRKRLRKFRNKIKEKLKKI	5.3979
2	GLRKALRKFRNKIKEALKKI	5.6990
3	GLRKRLRKARNKIKEKLKKI	5.3979
4	GLRKRLRKFRNKIKQKLKKI	5.3979
5*	FLGGLMKAFPALICAVTKKC	5.1549
6	FLGGLFKLVPSVICAVTKKC	4.9031
7	FLGGLMKIIPAAFCAVTKKC	5.0458
8	AALRGCWTKSIPPKPCPGKR	4.3354
9*	FLPIIAGMAAKVICAITKKC	4.3010
10	FFPIIAGMAAKVICAITKKC	4.8861
11	IASKVANTVQKLKRKAKNAV	4.6021
12	PRPPRLPRPRPRPLPFPRPG	4.7423
13	FLPFLLSALPKVFCFFSKKC	4.7959
14	FLPLLLSALPSFLCLVFKKC	4.4815
15*	GRFRRLRKKTRKRLKKIGKV	4.4949
16*	PRLPPRIPPGFPPRFPPRFP	5.3010
17	GLRRALLRLLRSLRRLLLRA	5.0969
18	LAKRRVLTLLRQLRRVSPSS	4.8539
19*	KRFWQLVPLAIKIYRAWKRR	5.6990
20*	PMLRVRLASHLRKLRKRLLR	5.2041
21	KIAKVALKALKIAKVALKAL	5.8239
22	KIAKVALKALKIAKGALKAL	6.1249
23	FRIRVRVFKRIVQRIKDFLR	6.0000
24	FRIRVRVAKKFGKAFVGEIM	5.0969
25*	KKRYKKKYKAYKPYKKKKKF	4.9031
26	SPRRRTPSPRRRRSQSPRRR	4.6021
27	RPRRRATTRRRITTGTRRRR	4.9031
28	RRLTLRQLLGLGSRRRRRSR	4.6021
29	GRRGPRRANQNGTRRRRRRT	4.6021
30*	WRRRYRRWRRRRRWRRRPRR	5.5045
31	IVPFLLGMVPKLVCLITKKC	4.1938

*Represent the test set

**Table 2:** Representative skeletons and molecular structures of antibacterial peptides targeted on *Staphylococcus aureus* and the inhibitory affinity pIC_50_ values

Compound	Sequence	pIC_50_(μM)
1	GLRKRLRKFRNKIKEKLKKI	5.3979
2	GLRKALRKFRNKIKEALKKI	5.6990
3	GLRKRLRKARNKIKEKLKKI	5.0969
4	GLRKRLRKFRNKIKQKLKKI	5.3979
5*	AALKGCWTKSIPPKPCSGKR	4.9318
6	AALRGCWTKSIPPKPCSGKR	5.2366
7	AALRGCWTKSIPPKPCPGKR	5.2366
8	SALVGCWTKSYPPNPCFGRG	4.9318
9*	SALVGCWTKSWPPKPCFGRG	4.6383
10	GRFRRLRKKTRKRLKKIGKV	5.2218
11*	KLLLKLKLKLLKGWKRKRFG	5.3979
12*	GAPKGCWTKSYPPQPCFGKK	4.7258
13	FFFHIVKGLFHAGRMIHGLV	5.9031
14	RPRRRATTRRRITTGTRRRR	4.3010
15*	RRLTLRQLLGLGSRRRRRSR	5.5045
16	WRRRYRRWRRRRRWRRRPRR	5.8069
17*	KIAKGALKALKIAKVALKAL	4.4949
18	KIGKALGKALKALGKALGKA	4.7959
19*	KIALKALKALKALGKALKAL	5.3979
20	GLYNFIKVLGRTVFGLYKQF	4.7959
21	GILSKLGKALKKAAKHAAKA	5.0969
22*	CKILSKTIKCRIPCGRRKEY	5.5229
23	GLLDFLKAAGKGLVSNLLEK	4.8239
24	YYHFWHRGVTKRSLSPHRPR	5.2218
25	KIGVLKKYFKIGALIKAIIK	5.0969
26	KKKFIYIVLALIKGAIIKKG	4.1938
27	KGKKGVIIAILLFAIIYKKK	3.8928
28	LKKLKQLLGKLSEFAAAFVA	4.4949
29	GQLNKFIKKAQRKFHEKFAK	3.8928
30	KVFKSVVKLLEKTVLKKFSK	4.1938
31*	KAAKTVFKLFKLQAKRAIEA	3.8928
32*	WCRRYRVLVRGVLVRYRRCW	5.3979
33	FLREFHKWIERVVGWLGKVF	4.4949
34	RQYMRQIEQALRYGYRISRR	4.0000
35	GSKKPVPIIYCNRRGKCQRM	4.9830
36	GSKKPVPIIYCNRRTKCQRM	4.6819
37	VGKTWIKVIRGIGKSKIKWQ	5.5528
38	KIAKVALKALKIAKVALKAL	5.8239
39	KIAKVALKALKIAKGALKAL	5.5229
40	FIVPSIFLLKKAFCIALKKC	5.0969

*Represent the test set

In addition, the structures of these peptides are built by the “Biopolymer” module in Sybyl software, which are further optimized using the Tripos Force Field and Gasteiger–Hückel charges (Clark et al. 1989; Gasteiger and Marsili 1980) with an energy charge of 0.05 kcal/mol·Å, and the maximum iteration coefficient of 100 (Joshi et al. 2016). Furthermore, the spatial structure and single-point calculation of amino acids are optimized by Gaussian’s B3LYP/6-31G** theory using Berny’s energy gradient method and generalized gradient approximation method at the DFT level.

### 2D-QSAR analysis

#### Descriptors generation and sequence characterization

The optimal conformations of the employed amino acids are imported into Dragon software (http://www.talete.mi.it/index.htm) to calculate related molecular descriptors, comprising 41 Randic molecular profiles, 150 RDF descriptors, 99 WHIM descriptors, 74 Geometrical descriptors, and 197 GETAWAY descriptors. In addition, to remove noise information and irrelevant variables, principal component analysis (PCA) is performed on the original variables, which is done by R software (Kim and Lee 2003). The first 2, 3, 4, 4 and 5 principal components of each type of descriptors would explain 97.25%, 81.24%, 81.06%, 83.76% and 84.65% of the variance of the original data matrix, respectively, suggesting that the selected principal components are sufficient to express the information of the original variable. Additionally, these principal components are used to replace the original variables as new amino acid descriptors for following QSAR studies. The new descriptors for the 20 basic amino acids are listed in Table 3.

**Table 3:** New descriptors for 20 basic amino acids

Amino acid	Scoring vector																	
	1	2	3	4	5	6	7	8	9	10	11	12	13	14	15	16	17	18
Ala	– 1.15	1.12	– 1.10	0.06	– 0.77	– 1.59	– 0.56	0.92	0.78	– 1.24	0.15	0.21	– 0.01	– 1.43	– 0.89	0.08	0.56	0.51
Arg	2.33	2.47	1.62	3.60	0.73	1.90	– 0.38	– 0.83	– 0.01	1.72	1.18	– 2.19	– 0.48	1.29	– 1.95	– 0.46	0.16	– 0.76
Asn	– 0.32	– 0.50	– 0.58	0.54	– 0.34	– 0.15	– 0.26	– 0.40	– 2.42	– 0.36	0.36	– 0.30	– 0.60	– 0.38	0.48	0.06	– 1.75	– 0.20
Asp	– 0.75	0.14	– 0.67	– 0.27	0.69	– 0.80	1.88	– 1.00	1.08	– 0.87	– 1.37	– 0.37	– 0.48	– 0.43	1.17	0.78	– 1.70	1.22
Cys	– 0.72	0.21	– 0.89	0.06	– 0.71	– 0.79	– 1.26	0.38	– 0.31	– 0.76	1.12	1.45	1.04	– 1.20	1.16	1.34	1.52	– 1.87
Gln	0.31	– 0.98	– 0.07	0.23	0.24	0.61	– 0.38	– 0.59	– 0.89	0.18	0.56	– 0.80	– 0.71	0.36	– 0.19	– 0.09	– 1.55	– 0.64
Glu	0.32	– 0.99	– 0.47	0.07	– 0.31	0.46	– 0.17	– 1.51	0.68	0.03	0.43	– 0.62	– 0.98	0.17	0.09	0.25	– 1.92	– 0.28
Gly	– 1.27	1.39	– 1.28	0.24	– 1.47	– 1.53	– 1.83	0.05	1.12	– 1.49	1.02	0.68	– 0.42	– 2.01	– 2.20	1.05	0.00	0.07
His	0.30	– 1.20	0.02	– 0.11	– 0.65	0.51	– 0.13	– 0.04	0.42	0.52	0.38	1.12	– 2.42	0.47	0.75	0.23	– 0.09	0.26
Ile	– 0.64	– 0.07	– 0.12	– 0.58	2.71	– 0.68	2.10	0.35	– 0.29	– 0.27	– 2.04	– 0.93	0.70	0.37	1.02	– 1.54	0.39	0.03
Leu	– 0.24	– 0.64	0.14	– 0.75	1.56	0.22	0.53	– 0.17	1.93	– 0.02	– 0.35	– 0.93	1.17	0.38	– 0.01	– 1.79	0.15	– 0.53
Lys	1.27	0.18	0.76	0.60	– 0.58	1.24	– 0.61	– 0.45	0.29	0.90	0.86	– 1.16	0.81	0.85	– 1.52	– 1.12	0.37	– 0.35
Met	0.48	– 0.78	0.03	0.04	– 0.58	0.67	– 0.72	– 0.30	– 1.03	0.30	1.37	0.72	2.37	0.28	0.83	0.96	0.54	– 2.25
Phe	0.80	– 1.14	1.32	– 1.23	– 0.60	0.80	– 0.17	0.58	0.75	1.03	– 0.16	1.20	0.73	1.01	0.21	0.69	0.49	0.31
Pro	– 0.84	0.36	– 0.67	– 0.39	0.33	– 0.77	0.34	– 0.95	– 0.99	– 0.77	– 0.70	0.06	– 0.14	0.59	0.60	0.25	1.36	2.26
Ser	– 0.83	0.41	– 0.92	0.45	– 0.68	– 1.05	– 1.12	0.83	– 0.43	– 0.93	0.55	0.34	– 0.67	1.16	0.58	0.40	0.10	0.30
Thr	– 0.75	0.21	– 0.48	– 0.05	0.65	– 0.59	0.50	– 0.03	0.11	– 0.71	– 0.38	– 0.15	– 0.04	0.62	0.42	0.89	0.26	0.18
Trp	0.81	– 1.52	2.09	– 1.15	– 0.43	1.05	0.89	3.28	– 0.49	2.01	– 1.80	1.68	– 0.04	1.83	0.27	1.83	0.84	1.29
Tyr	1.71	0.94	1.79	– 0.95	– 0.91	1.25	0.00	0.39	0.75	1.42	0.06	0.72	– 0.38	1.20	0.12	0.69	0.09	0.38
Val	– 0.84	0.38	– 0.52	– 0.41	1.11	– 0.76	1.33	– 0.49	– 1.03	– 0.67	– 1.23	– 0.74	0.54	0.37	1.00	1.43	0.91	0.05

The new descriptors are used to characterize the structure of each active peptide in the peptide library. For example, a peptide containing an amino acid residues can be characterized by 18 × n variables. If the characterized peptides contain different number of amino acids, different number of independent variables will be derived. In addition, data normalization is also performed to unify the data. The number of amino acids of AMPs employed in this study is the same, thus, this operation is omitted.

#### Multiple linear regression (MLR) method

MLR is a supervised method that can establish a mathematical relationship between molecular descriptors (independent variables) and biological activities (dependent variable) (Aiken et al. 2003), which is a traditional and standard approach for multivariate data analysis. In MLR analysis, the structural characteristics to the activity can be described as follows:

1 $$\log IC_{50} = b_{0} + b_{1} X_{1} + b_{2} X_{2} + \ldots b_{n} X_{n}$$

where *b*_0_ is the intercept; *b*_1_, *b*_2_ and *b*_*n*_ are regression coefficients; *n* is the number of descriptors employed in the equation; *X*_*1*_, *X*_*2*_ and *Xn* are independent variables which are used to describe the chemical structure of the peptide.

#### Partial least squares regression (PLSR) method

PLSR is an effective technique for constructing the relationship between the properties (matrix *Y*) of a compound and its structure (matrix *X*), which can handle strong correlated or noisy X variables (Wold 1994). In addition, PLSR is an expansion of MLR method, which extends MLR without imposing restrictions employed in discriminant analysis, principal component regression and canonical correlation (Thombare et al. 2012). PLSR tries to find the multidimensional direction in the X space that would explain the maximum multidimensional variance direction in the Y space. The PLSR model can be developed when the matrix of predictors has more variables than observations, and when multi-collinearity among X values is presented. Furthermore, the detailed parameters for PLSR method can be found in references (Geladi and Kowalski 1986; Rosipal and Krämer 2005).

#### Support vector regression (SVR) method

Support vector machine (SVM) as a supervised algorithm has been mainly used for pattern recognition classification (Burges 1998; Sadeghi et al. 2013). Recently, SVM has been used to solve non-linear regression estimation for the introduction of ε-insensitive loss function (Drucker et al. 1997), which is named as SVR method. The idea of SVR is that the non-linear vectors are mapped to a high-dimensional feature space using one of the kernel functions (Cortes and Vapnik 1995; Shawe-Taylor and Cristianini 2000; Smola and Schölkopf 2004).

For constructing SVR model, the activities and chosen molecular descriptors are denoted as *y*_*i*_ and *x*_*i*_, and the correlation relationship is expressed as *y*_*i*_ = *f*(*x*_*i*_). Different kernels (linear kernel, radial basis function-RBF kernel, sigmoid kernel, and polynomial kernel) are tried to describe non-linear transformations of higher dimensional space. In addition, the credibility of SVR is also relied on other factors, such as capacity parameter *C*, *ε* of *ε*–insensitive loss function and corresponding parameters (Vapnik 1998).

### 3D-QSAR analysis

#### Molecular alignment

**Fig. 1:**
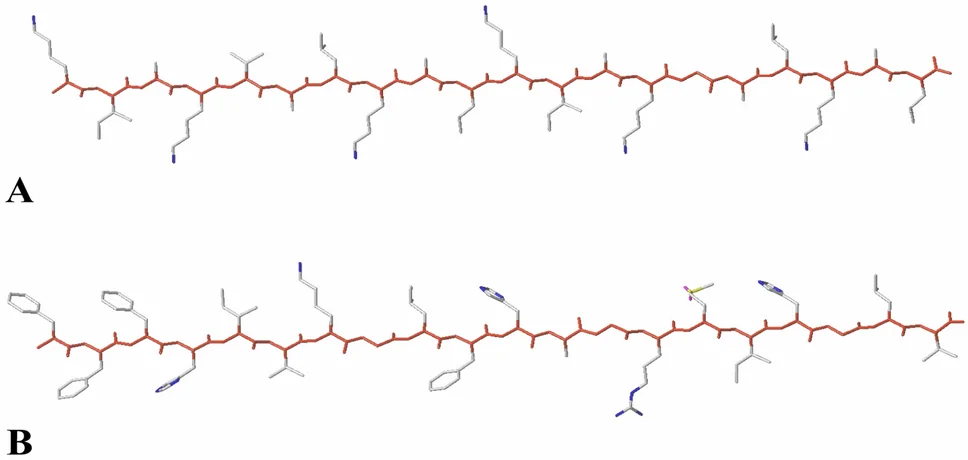
**A** Peptide 22 used as a template for *E.coli*. The common substructure is shown in red. **B** Peptide 13 used as a template for *Staphylococcus aureus*. The common substructure is shown in red

**Fig. 2:**
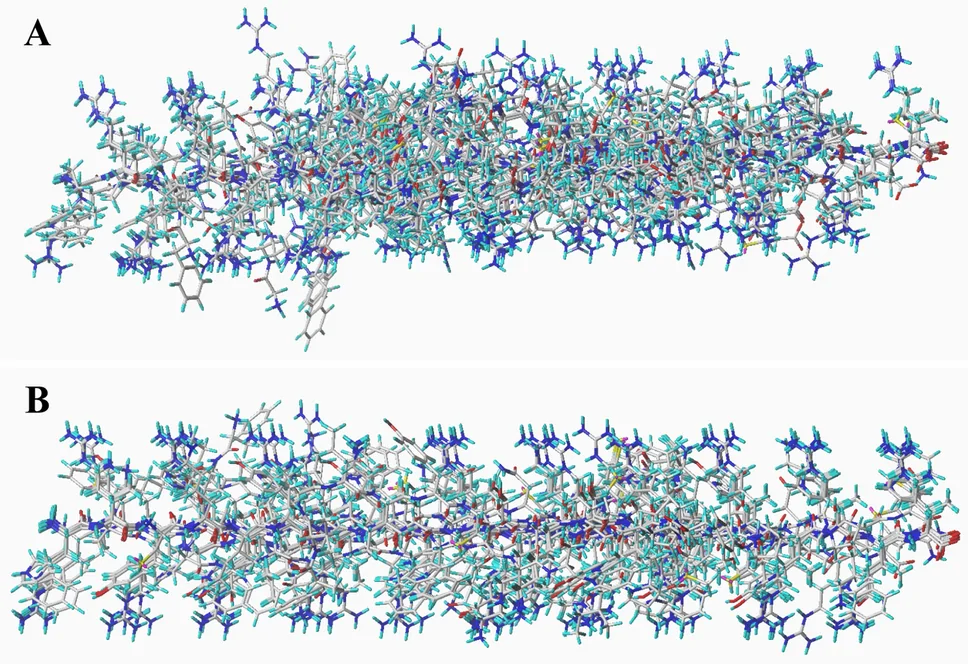
**A** the alignments for antibacterial peptides targeted on *E.coli* from the template ligand-based alignment. **B** the alignments for antibacterial peptides targeted on *Staphylococcus aureus* from the template ligand-based alignment

In 3D-QSAR studies, molecular alignment is one of the most significant factor that would affect the quality of the model (Liu et al. 2015; Wang et al. 2015). In the present work, the template ligand-based alignment is adopted which the most active peptides (peptide 22 for *Escherichia coli*; peptide 13 for *Staphylococcus aureus*) are employed as the template, and the remaining peptides are aligned on them depending on the common substructures (as shown in Fig. 1, the red atoms represent the common substructures). Furthermore, the results of alignment are shown in Fig. 2.

#### 3D-QSAR studies

In this work, the 3D-QSAR models were developed using CoMFA and CoMSIA approaches. A 3D cubic lattice with grid spacing of 2.0 Å is generated to calculate the CoMFA and CoMSIA fields. For CoMFA analysis, steric field based on Lennard–Jones potential and electrostatic field based on Coulombic potential with a distance-dependent dielectric at each grid point are computed using a sp^3^ carbon atom probe with a van der Waals radius of 1.52 Å and a charge of + 1.0 using default parameters. Additionally, the minimum column filtering is set to 2.0 kcal/mol to reduce noise and improve efficiency. The steric and electrostatic contributions are truncated at a default value of 30.0 kcal/mol. Furthermore, the other parameters are set to the default settings as described in literature (Mittal et al. 2009). For CoMSIA model, five physicochemical properties (steric, electrostatic, hydrophobic, hydrogen bond donor and hydrogen bond acceptor) are calculated using the same probe atom with radius 2.0 Å, + 1.0 charge, and hydrophobic + 1, hydrogen bond donor + 1 and hydrogen bond acceptor + 1. The attenuation factor and column filtering are set to 0.3 and 2.0 kcal/mol, respectively. In addition, a Gaussian function is applied to calculate the distance between the probe atom and each atom of the peptide.

To derive reliable 3D-QSAR models, PLS regression analysis is carried out to correlate the activities to CoMFA and CoMSIA fields. Initially, leave-one-out (LOO) cross-validation is performed to determine the cross-validated correlation coefficient R^2^_cv_ and the optimum number of principal components (Nc). Then non-cross-validation is performed with the generated Nc to obtain the non-cross-validated correlation coefficient R^2^_ncv_, standard error of estimation (SEE), F value and contributions of each field.

To further validate the robustness of the derived 3D-QSAR models, the activities of the test set peptides are predicted using the following formula:

2 $$R_{{{\mathrm{pred}}}}^{2} = \frac{{(SD - {\mathrm{PRESS}})}}{SD}$$

where *SD* is the sum of squared deviations between the activities of the test set and the mean activity of the training set, and PRESS is the sum of squared deviation between the actual and predicted activity of each peptide in the test set (Nandi and Bagchi 2010).

### Applicability domain analysis

The reliability of the developed QSAR models depends on the prediction ability for novel peptides. The constructed models are regarded as valid only when compounds fall within the applicability domain, therefore, the application domain was calculated in the present work for 2D-QSAR models and 3D-QSAR models, which is derived by using the following approach: https://dtclab.webs.com/softwaretools or https://teqip.jdvu.ac.in/QSAR_Tools/.

## Results and discussion

### 2D-QSAR results

#### The results of MLR for *Escherichia coli*

Generally, the correlation between molecular descriptors and activities is the most significant means of structure–activity relationship study (Hall and Kier 1999). Therefore, the equation should possess the least number of molecular descriptors to obtain the best model. The best 2D-QSAR model using MLR method is presented as follows:

3 $$\begin{gathered} {\mathrm{pIC}}_{{{5}0}} = {5}.{218} - 0.{356} \times {\mathrm{X359}} - 0.{258} \times {\mathrm{X3}}0{9} + 0.{3}0{6} \times {\mathrm{X289}} \hfill \\ {\mathrm{n}}_{{{\mathrm{training}}}} = {23};{\text{ n}}_{{{\mathrm{test}}}} = {8};{\text{ R}}^{{2}} = 0.{718};{\text{ Q}}^{{2}} = 0.{6725};{\text{ F}} = {16}.{117};{\text{ SEE}} = 0.{2897};{\text{ SEP}} = 0.{4143} \hfill \\ \end{gathered}$$

**Fig. 3:**
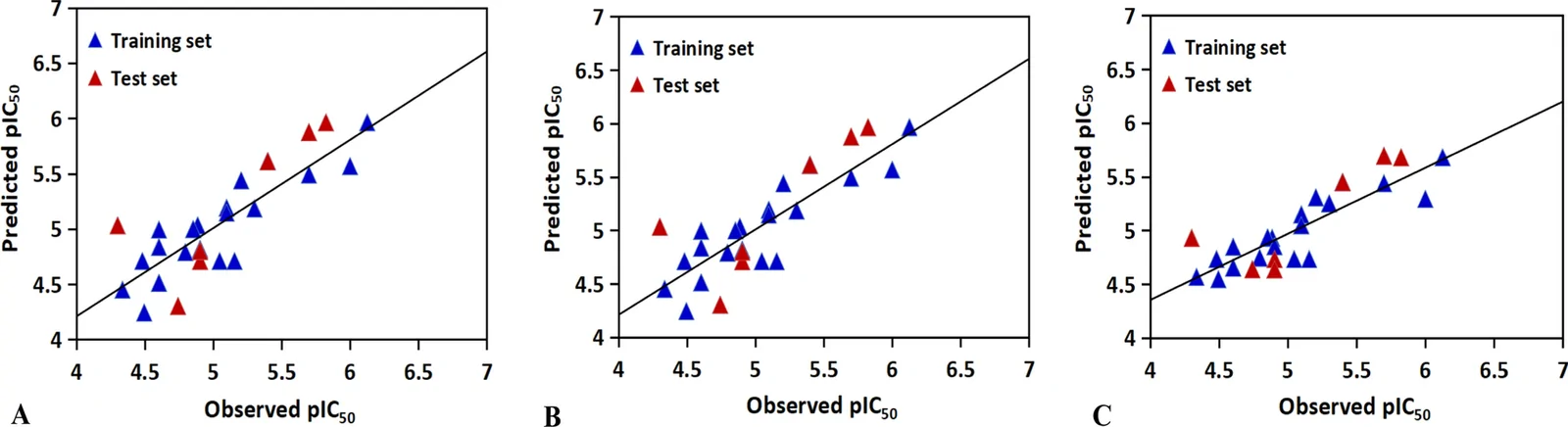
Experimental *vs* predicted pIC_50_ values of antibacterial peptides targeted for *Escherichia coli* by **A** the MLR model; **B** the PLSR model; **C** the SVR model

The above MLR model would explain 71.8% of the variance (adjusted coefficient of variation) with low standard error of SEE = 0.2897, indicating that the MLR model has good internal predictive power. The F value of 16.117 suggests the statistical significance level for the model. In addition, the external predictive ability of the model is also powerful with Q^2^ of 0.6725 and SEP of 0.4143. The resulting model is illustrated in Fig. 3A, where the relationship between the actual activities and the predicted values is presented, all the data show that the model is predictive from both internal and external aspects. For the MLR equation, the standardized coefficient indicates that the most significant descriptor is X359 (correspond to GETAWAY descriptors for the twentieth amino acid), its negative coefficient may be interpreted as that low value of this descriptor can lead to the increased activity. The GETAWAY descriptors (Consonni et al. 2002) are derived from the representation of molecular structure according to an influence matrix (H-GETAWAY) or influence-distance matrix (R-GETAWAY). In addition, descriptors R1e + , RTe + , RTu, RTv, and RTu + (Table 4) also show higher correlation with the activities. By analyzing the relevant parameters, we found that the antimicrobial activities can be improved when the atomic Sanderson electronegativities and van der Waals volumes of the twentieth amino acid are decreased. The second contributor descriptor is X289, positively correlated with the activity. The R3e descriptor is mainly involved in X289, further indicating that increasing the atomic Sanderson electronegativities of the sixteenth amino acid, the activity will be increased. Another significant factor X309 also has significant influence on the activity, which is corresponding to Randic molecular profiles. The Randic molecular profiles (Randic 1995) are computed from the geometric interatomic distance for all atoms from the atomic periphery. X309 is constituted mainly by molecular descriptors DP20 and SP20, demonstrating that the molecular profile and shape profile of the eighteenth amino acid are related to the activity of the peptide. All the above information may also be useful in a novel peptide design.

**Table 4:** Descriptors used in model construction

Symbol	Class	Meaning
R1e +	GETAWAY descriptors	R maximal autocorrelation of lag 1/weighted by atomic Sanderson electronegativities
Rte +	GETAWAY descriptors	R maximal index/ weighted by atomic Sanderson electronegativities
Rtu	GETAWAY descriptors	R total index/unweighted
RTv	GETAWAY descriptors	R total index/weighted by atomic van der Waals volumes
Rtu +	GETAWAY descriptors	R total index/unweighted
R3e	GETAWAY descriptors	R autocorrelation of lag 3/weighted by atomic Sanderson electronegativities
DP20	Randic molecular profiles	Molecular profile no. 20
SP20	Randic molecular profiles	Shape profile no. 20
SPAM	Geometrical descriptors	Average span R
ASP	Geometrical descriptors	Asphericity
MEcc	Geometrical descriptors	Molecular eccentricity
SPH	Geometrical descriptors	Spherosity
L2m	WHIM descriptors	2nd component size directional WHIM index/weighted by atomic masses
L2v	WHIM descriptors	2nd component size directional WHIM index/weighted by atomic van der Waals volumes
L2p	WHIM descriptors	2nd component size directional WHIM index/weighted by atomic polarizabilities
L2e	WHIM descriptors	2nd component size directional WHIM index/weighted by atomic Sanderson electronegativities
L2s	WHIM descriptors	2nd component size directional WHIM index/weighted by atomic electrotopological states
R2p	GETAWAY descriptors	R autocorrelation of lag 2/weighted by atomic polarizabilities

#### The results of PLSR for *Escherichia coli*

**Fig. 4:**
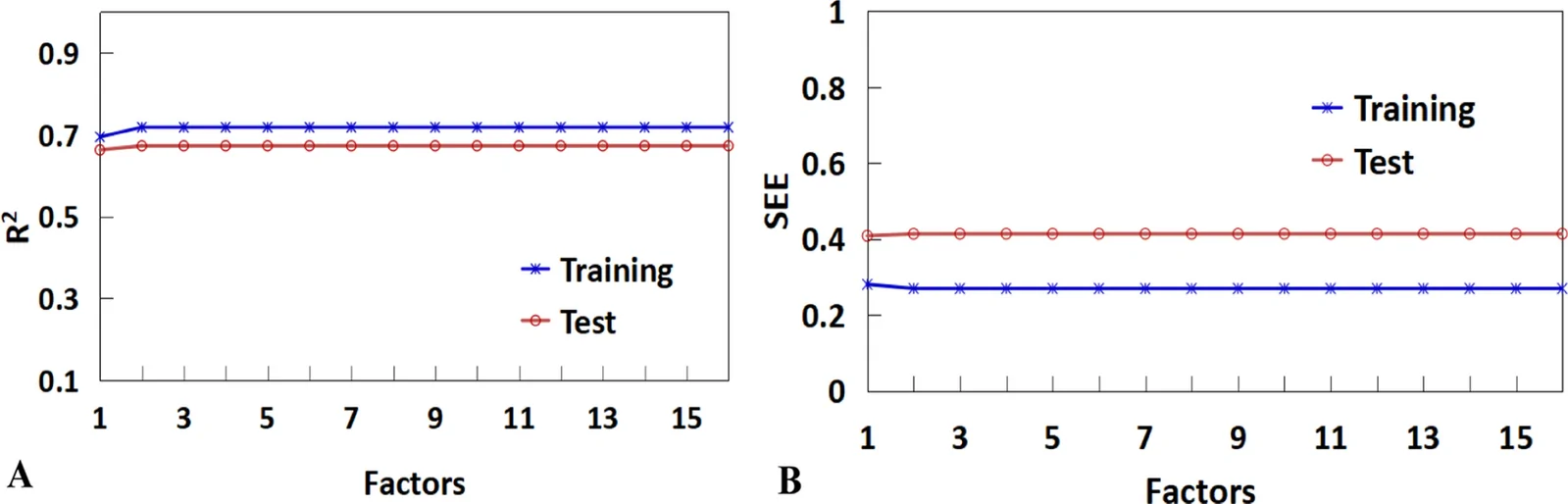
Trend of the statistical results of the PLSR models for antibacterial peptides targeted for *Escherichia coli* with vary latent factors based on the data sets

Based on the same dataset and molecular descriptors employed in the MLR model, PLSR model was also developed to predict the activity. For the PLSR model, the most important question is how many factors should be chosen. In the present work, when factors were set to three, an optimal model was derived (Fig. 4). The corresponding statistical correlation coefficients (R^2^ and Q^2^) are 0.7179 and 0.6725, respectively for the training set and the test set. Furthermore, the standard error is 0.2692 for the training set and 0.34134 for the test set. The predicted *versus* experimental activities based on PLSR is shown in Fig. 3B. These data indicate that the obtained PLSR model has good internal and external predictive power.

#### The results of SVR for *Escherichia coli*

The same descriptors selected in MLR model were also used as the input parameters to construct SVR model. As shown in Table 5, when type = Radial basis function and Epsilon = 0.1, the best model is derived, the other parameters are set as shown in Table 6.

**Table 5:** The results of SVR with different kernel function under the same parameters

Pseudo-r^2^	Linear kernel (0.1)	Linear kernel (0.05)	RBF kernel (0.1)	RBF kernel (0.05)	Sigmoid kernel (0.1)	Sigmoid kernel (0.05)	Polynomial kernel (0.1)	Polynomial kernel (0.05)
Training set	0.6921	0.6887	0.7007	0.6839	0.4421	0.4249	0.0000	0.0000
Test set	0.5776	0.5801	0.7406	0.7432	0.1859	0.0944	– 0.0020	0.0006

**Table 6:** Selected parameters of the SVR algorithm

Parameters	Selected values
Type	Radial basis function-SVR
Epsilon	0.1
Kernel type	Radial basis function
Degree (poly)	3
Gamma in kernel function (poly/rbf/sigmoid)	0
Coef0 in kernel function (poly/sigmoid)	0
Tolerance of termination criteria	0.001
*C* (Complexity Cost)	1
Use shrinking heuristics	1

The statistical characteristics for the data set are as follows: R^2^ = 0.7007; Q^2^ = 0.7406, indicating that the SVR model also has satisfactory robustness and predictive ability. In addition, the predicted values are consistent with the experimental values, as indicated in Fig. 3C.

Overall, the performance of SVR model for antibacterial peptides targeted *Escherichia coli* is much better than  those of MLR and PLSR models, indicated by the correlation R and standard error of estimation. Thus, the SVR model can be used to screening and designing novel antibacterial peptides with improved activities.

#### The results of MLR for *Staphylococcus aureus*

4 $$\begin{gathered} {\mathrm{pIC}}_{{{5}0}} = {4}.{766} + 0.{286} \times {\mathrm{X12}}0 + 0.{34}0{8} \times {\mathrm{X261}} + 0.{686} \times {\mathrm{X18}} - 0.{148} \times {\mathrm{X84}} - 0.0{5}0 \times {\mathrm{X111}} \hfill \\ {\mathrm{n}}_{{{\mathrm{training}}}} = {3}0;{\text{ n}}_{{{\mathrm{test}}}} = {1}0;{\text{ R}}^{{2}} = 0.{575};{\text{ Q}}^{{2}} = 0.{5645};{\text{ F}} = {8}.{837};{\text{ SEE}} = 0.{4}0{34};{\text{ SEP}} = 0.{2519} \hfill \\ \end{gathered}$$

**Fig. 5:**
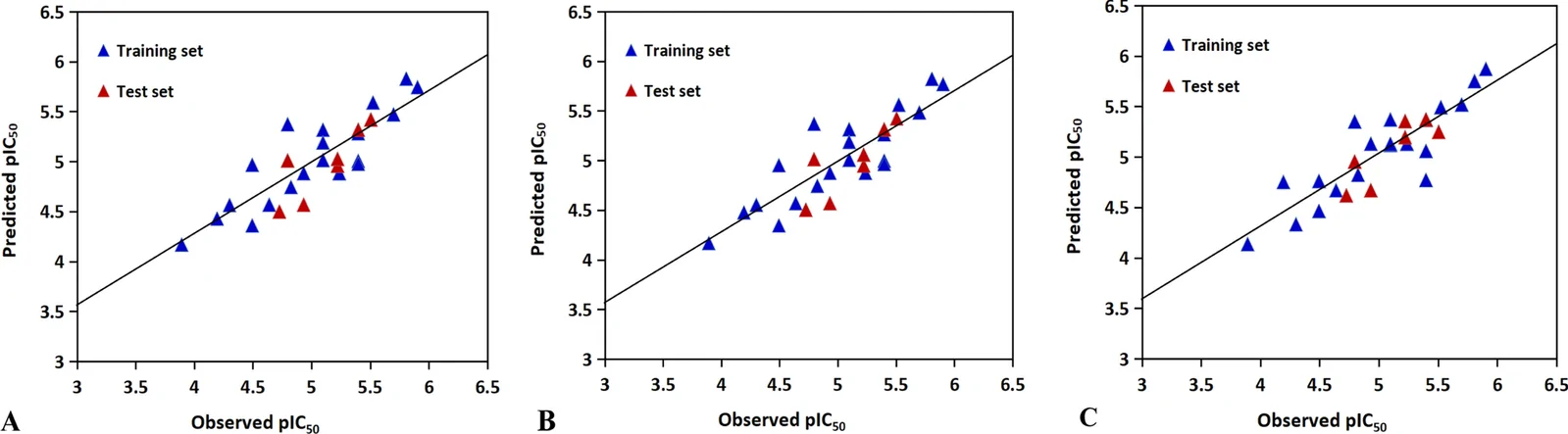
Experimental *vs* predicted pIC_50_ values of antibacterial peptides targeted for *Staphylococcus aureus* by **A** the MLR model; **B** the PLSR model; **C** the SVR model

The optimal MLR model for the training set based on the molecular descriptors (X120, X261, X18, X84 and X111) has a correlation coefficient of 0.575, a standard error of estimation of 0.4034. The test set predicted by the MLR model gives a correlation coefficient of 0.5645 and a standard error prediction of 0.2519. The plot of actual activities *versus* predicted activities is shown in Fig. 5A.

#### The results of PLSR for *Staphylococcus aureus*

**Fig. 6:**
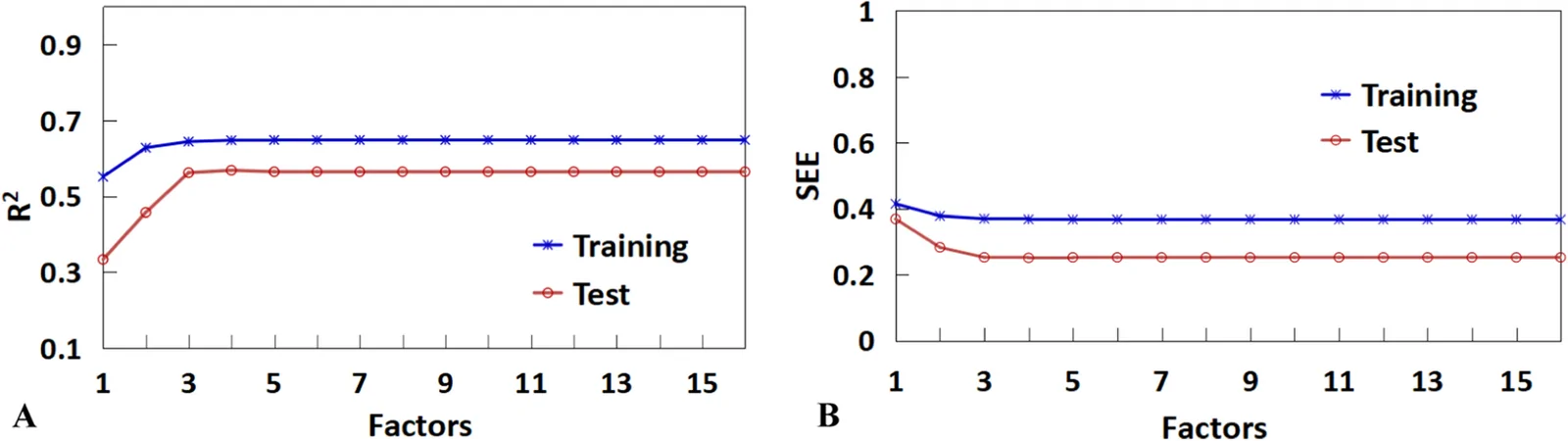
Trend of the statistical results of the PLSR models for antibacterial peptides targeted for *Staphylococcus aureus* with vary latent factors based on the data sets

The same descriptors were also selected for constructing the PLSR model (also using the same training set). The PLSR model was chosen with four components whose parameters are indicated in Fig. 6. The correlation calibration variance for the dependent variable is 64.73% and the standard error of estimation is 0.3674. The explained prediction variance of the independent variable is 56.83% and the standard error of prediction is 0.2505. Furthermore, the predicted activities *vs* actual activities are  shown in Fig. 5B, displaying the goodness of the fit for the model.

#### The results of SVR for *Staphylococcus aureus*

The best SVR model listed in Tables 7 and 8 shows a correlation coefficient of 0.6999. The test set predicted by using the constructed SVR model  achieves a correlation coefficient of 0.7418. The correlation diagram with experimental values *versus* calculated values is shown in Fig. 5C.

**Table 7:** The results of SVR with different kernel function under the same parameters

Pseudo-r^2^	Linear kernel (0.1)	Linear kernel (0.05)	RBF kernel (0.1)	RBF kernel (0.05)	Sigmoid kernel (0.1)	Sigmoid kernel (0.05)	Polynomial kernel (0.1)	Polynomial kernel (0.05)
Training set	0.6250	0.6338	0.6984	0.6999	0.1665	0.1720	0.0000	0.0000
Test set	0.4387	0.5125	0.7616	0.7418	– 0.9042	– 0.9366	0.0132	– 0.0071

**Table 8:** Selected parameters of the SVR algorithm

Parameters	Selected values
Type	Radial basis function-SVR
Epsilon	0.05
Kernel type	Radial basis function
Degree (poly)	3
Gamma in kernel function (poly/rbf/sigmoid)	0
Coef0 in kernel function (poly/sigmoid)	0
Tolerance of termination criteria	0.001
C (Complexity Cost)	1
Use shrinking heuristics	1

According to the above data, we can draw the conclusion that the prediction of SVR model is better than those of MLR and PLSR model. In addition, there are five important factors: X120, X261, X18, X84 and X111. In this system, the greatest contributor is X18, and have a positive correlation with the activities. X18 descriptor belongs to the GETAWAY descriptors. In this series of GETAWAY descriptors, R2p plays an important role in affecting the activity of the peptide (corresponding to the first amino acid in the N-terminal), therefore, increasing the polarizabilities of this amino acid would enhance the activity. Furthermore, the other WHIM descriptors, i.e., L2m, L2v, L2p, L2e and L2s also provide a major force in improving the activity. Its positive coefficient may be interpreted as that higher value of the atomic masses, van der Waals volumes, electronegativities, and polarizabilities (for the fifteenth amino acid) can lead to increased activity. The positive contribution of Geometrical descriptors (SPAM, ASP, MEcc, and SPH) towards the prediction of peptide activity might be enhanced by increasing the value of the descriptors for the seventh amino acid. On the contrary, the negative contribution of the Geometrical descriptors for the X84 factor suggests that the activity would be improved by decreasing the value of the descriptors for the fifth amino acid. Additionally, the low DP20 and SP20 values, as the randic molecular profiles, positively influence the antimicrobial activity, thus pointing toward the need for the larger groups at the seventh amino acid.

### 3D-QSAR results

The CoMFA and CoMSIA models based on the training set were employed to investigate the existence of any correlation between chemical structures and activities. For these models, high cross-validated correlation coefficient (*R*^2^_cv_) and non-cross-validated correlation coefficient (*R*^2^_ncv_) are considered as credible models. The statistical parameters of CoMFA and CoMSIA models are listed in Table 9.

**Table 9:** Statistical data of optimal QSAR models

	Results for *Escherichia coli*		Results for *Staphylococcus aureus*	
Parameters	CoMFA	CoMSIA	CoMFA	CoMSIA
*R*^2^_cv_	0.537	0.512	0.607	0.556
*R*^2^_ncv_	0.822	0.980	0.970	0.969
SEE	0.232	0.085	0.111	0.112
F	46.240	166.016	153.531	151.151
*R*^2^_pred_	0.5401	0.5675	0.5883	0.5371
SEP	0.375	0.418	0.399	0.424
Nc	2	5	5	5
*Field contribution*				
S	0.451	–	0.408	–
E	0.549	0.522	0.592	0.711
H	–	0.478	–	–
D	–	–	–	–
A	–	–	–	0.289

*R*^2^_cv_ = cross-validated correlation coefficient using the leave-one-out methods

*R*^2^_ncv_ = Non-cross-validated correlation coefficient, *SEE*  Standard error of estimate, *F*   Ratio of *R*^2^_ncv_ explained to unexplained = R^2^_ncv_/(1-R^2^_ncv_)

*R*^2^_pred_ = Predicted correlation coefficient for the test set of compounds, *SEP*   Standard error of prediction, *N*_*C*_   Optimum number of principal components, *S*   steric, *E*   electrostatic, *H*   hydrophobic, *D*   H-bond donor, *A*   H-bond acceptor

#### CoMFA and CoMSIA statistical results for *Escherichia coli*

**Fig. 7:**
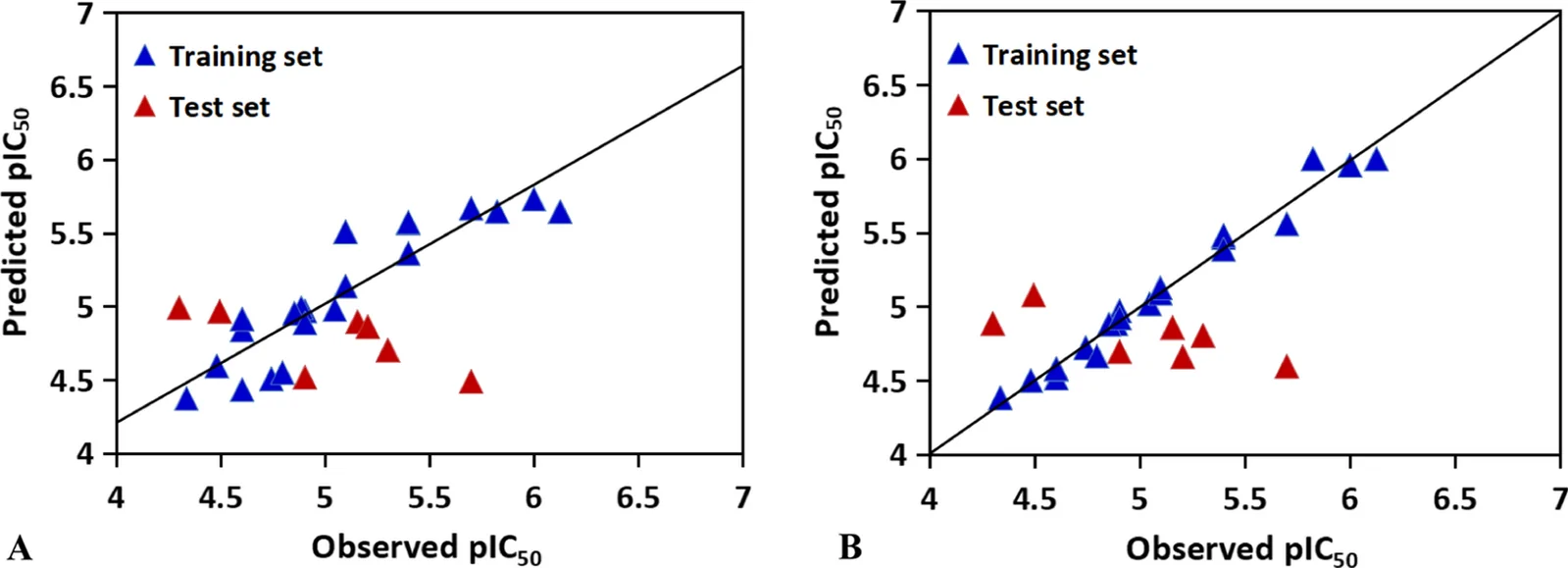
The correlation plots of the actual versus the predicted pIC_50_ values using the training set based on the CoMFA and CoMSIA models obtained from the activity for *Escherichia coli* antibacterial peptides. Graphs of the predicted versus the experimental pIC_50_ values of the optimal models. **A** CoMFA model. **B** CoMSIA model

For CoMFA model, the PLS analysis gives cross-validated *R*^2^_cv_ of 0.537 with optimum number of components (Nc) of 2, standard error of estimation (SEE) of 0.232, non-cross validated coefficient (*R*^2^_ncv_) of 0.822, F value of 46.240. All these data suggests that reliable 3D-QSAR model has been successfully constructed. Moreover, the external predictive ability of the model is evaluated using the test set with the predicted correlation coefficient (*R*^2^_pred_) of 0.5401, suggesting the satisfactory predictive ability of the model. The contributions of the steric and electrostatic fields are 45.1% and 54.9%, respectively. Furthermore, the correlation of the actual and predicted values using the CoMFA model is shown in Fig. 7A, indicating that the predicted values are consistent with the experimental ones.

Different combinations of CoMSIA descriptors were also employed to generate models, as shown in Table S1. The combination of the electrostatic and hydrophobic descriptors yield the most robust CoMSIA model (*R*^2^_cv_ = 0.512, Nc = 5, *R*^2^_ncv_ = 0.980, SEE = 0.085, F = 166.016, SEP = 0.418, *R*^2^_pred_ = 0.5675, electrostatic contribution = 0.522, hydrophobic contribution = 0.478), validating the robustness and predictability of the CoMSIA model. The correlation of actual and predicted activities is plotted in Fig. 7B.

#### CoMFA and CoMSIA statistical results for *Staphylococcus aureus*

**Fig. 8:**
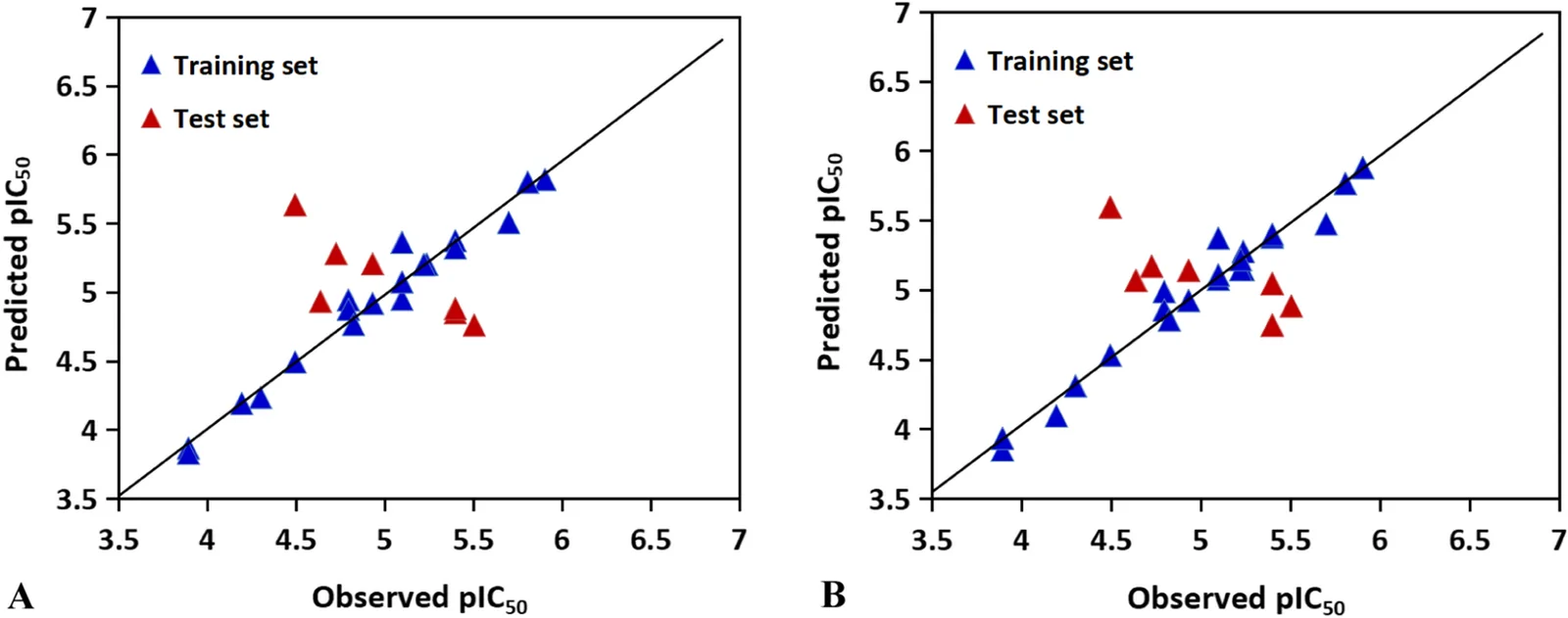
The correlation plots of the actual versus the predicted pIC_50_ values using the training set based on the CoMFA and CoMSIA models obtained from the activity for *Staphylococcus aureus* antibacterial peptides. Graphs of the predicted versus the experimental pIC_50_ values of the optimal models. **A** CoMFA model. **B** CoMSIA model

The results of statistical parameters for the CoMFA and CoMSIA models are denoted in Table 9. The cross-validated R^2^_cv_ is 0.607, the Nc is 5, the non-cross-validation coefficient R^2^_ncv_ of CoMFA model is 0.970, the standard error of estimation is 0.111, the F value is 153.531. Thus, the constructed model has reliable prediction ability and good fitting ability. Moreover, the contribution of the steric and electrostatic field is 40.8% and 59.2%, respectively, suggesting that the electrostatic groups would have more significant influence on the activity of the peptide. In addition, to validate the external capability of the CoMFA model, the activities of the test set peptides are predicted with R^2^_pred_ of 0.5883 produced. The scatter plot is drawn to describe the correlation between the experimental values and predicted values for the data set. As shown in Fig. 8A, all points are at or near the trend line, further illustrating that the model possesses good external prediction ability.

In the CoMSIA model, the combination of electrostatic and hydrogen bond acceptor fields performs well among the possible different field combinations (*R*^2^_cv_ = 0.556, Nc = 5, *R*^2^_ncv_ = 0.969, SEE = 0.112, F = 151.151) (Table S2). The contribution of electrostatic and hydrogen bond acceptor field is 71.1% and 28.9%, respectively, indicating that the electrostatic field contributes more to the model. This is consistent with the CoMFA model, and the information can be used to guide the modification of these peptides. The reliability and predictive power is verified with the peptides in the test set. The correlation between the predicted and experimental activities is shown in Fig. 8B. The predicted activities are in good agreement with the experimental ones with *R*^2^_pred_ of 0.5371, indicating the CoMSIA model is reliable.

### Contour analysis

The contour maps were produced as an informative tool to identify the effects of different fields (steric, electrostatic, hydrophobic, hydrogen bond donor and hydrogen bond acceptor) on the  activities of this series of peptides. The contour maps provide information about the physicochemical properties of peptides and represent the favorable (80%) and unfavorable (20%) areas in the peptide for the activity. Additionally, the most active peptides (peptide 22 for *Escherichia coli*, peptide 13 for *Staphylococcus aureus*) are used as templates for contour map analysis.

#### Contour maps for *Escherichia coli*

**Fig. 9:**
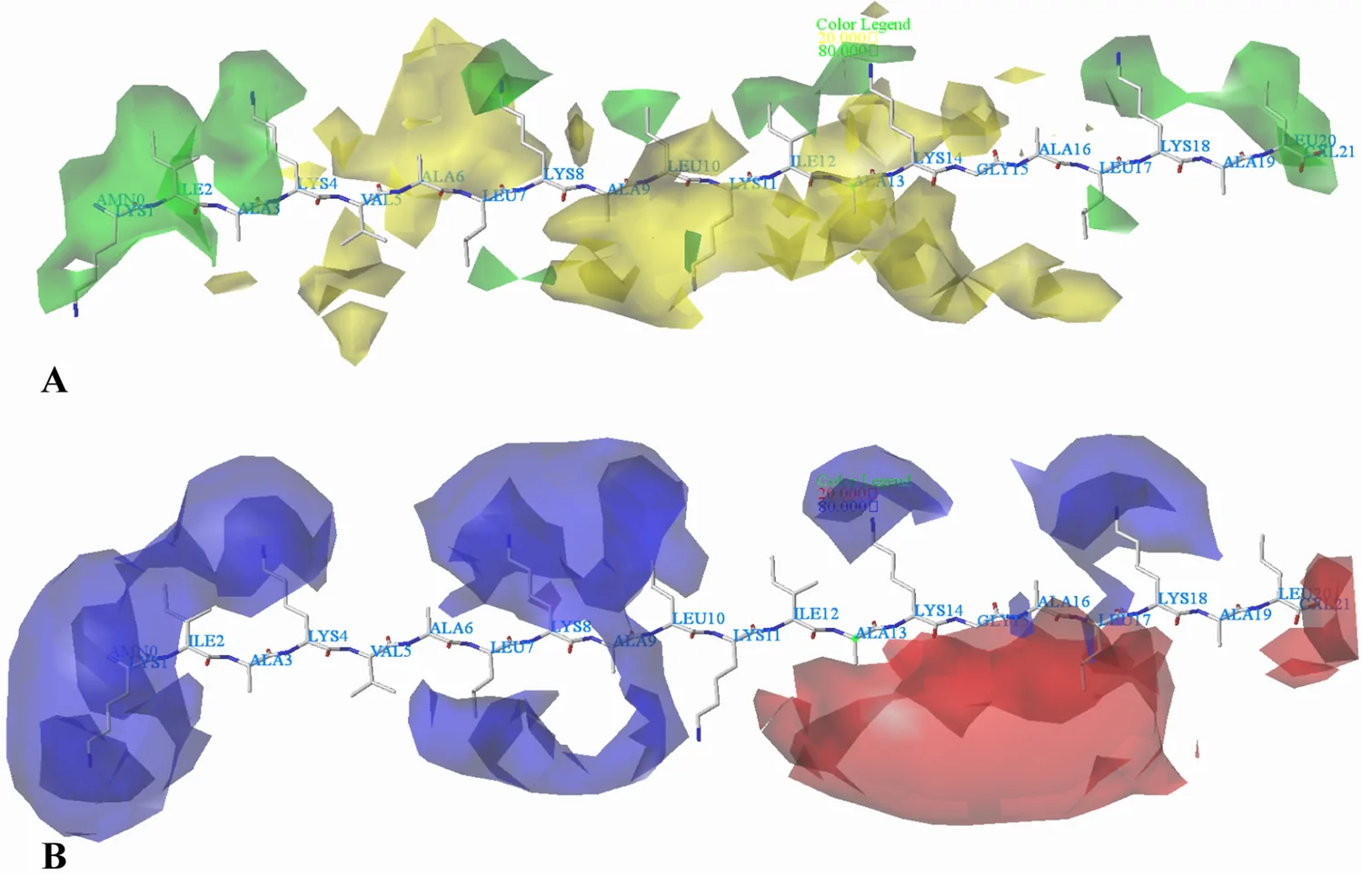
CoMFA StDev*Coeff contour plots for *Escherichia coli* in combination of peptide 22. **A** The steric contour map, where the green and yellow contours represent 80% and 20% level contributions, respectively. **B** The electrostatic contour map, where the blue and red contours represent 80% and 20% level contributions, respectively

In Fig. 9A, the contour map of the steric field of CoMFA model is displayed, the green contour maps denote the sterically favored regions and the yellow contour maps are the sterically disfavored regions. A green contour polyhedron located around the first amino acid (Lys) at the N-terminal suggests that appropriately bulky groups have favorable steric interactions. Peptide 27 and peptide 26 are taken for explanation, peptide 27 with Arg at this position has better activity than peptide 26 (Ser). A green contour besides the second amino acid (Ile) is consistent with the order of activities for peptide 9 (Leu) and peptide 10 (Phe), peptide 10 > peptide 9. There is a green region covering the fourth amino acid, implying that connecting to the steric bulk substituent is beneficial to the activity of the peptide. And this can be proved by the comparison of peptides 13 and 14. As the steric contour map connecting to Phe (peptide 13) enhances the activity when compared with peptide 14 (Leu). Several yellow contour maps appear near the fifth amino acid, suggesting that a substituent of minor groups would favor the activity. For example, peptide 2 with Ala shows more potent activity than peptide 1 with Arg at this position. A large yellow contour is projected to the sixth amino acid. This means that minor substituents at this position favor the activity. As example, peptide 6 has substituent Phe, and exhibits higher activity than peptide 7 (Met). At the seventh amino acid (Leu), a green contour map is observed, indicating that a bulky substituent would increase the activity. The eighth amino acid (Lys) is oriented within a green polyhedron, which indicates that bulky residues favor the activity (i.e. the activity of peptide 23 (Phe) is higher than peptide 24 (Ala)). The ninth amino acid (Ala) is projected into a large yellow contour map, illustrating that minor substituents at this area may increase the activity. A green contour extending over the tenth amino acid suggests that large groups are favorable. Therefore, modifications can be made at this position to improve the activity. There is a big yellow contour covering the eleventh amino acid (Lys), thus minor groups at this position can improve the activity. A comparison between peptide 5 and peptide 6 shows that as the larger Ser in peptide 6 replaces with a smaller Ala in peptide 5, biological activity increases. Therefore, modifications can be made at this area for the most active peptide. A green contour map is located around the twelfth amino acid (Ile), indicating that a large group is favorable. For example, the higher activity of 23 (Val) than 24 (Gly) reals that peptide 23 has an increased steric bulky substituent. We also observe that several yellow contour maps cover the thirteenth amino acid (Ala), which indicates that minor substitution group selection is required in this region. For instance, the activity of peptide 23 (Gln) is higher than peptide 24 (Lys). Around the fourteenth amino acid (Lys), there is a green contour, indicating that increasing the volume is conducive to improve the activity. For example, peptide 23 introduces Arg at this position, and the activity improves compared with peptide 24 (Ala). There is a yellow contour map at the fifth amino acid (Gly), illustrating small group is beneficial for the improvement of the activity, such as peptide 22 (Gly), which is more active than peptide 21 (Val). There is a green contour map at the seventeenth amino acid (Leu), suggesting that bulky groups at that position might be more beneficial for the activity. This is consistent with the following activity orders: 23(Asp) > 24(Gly). A green block appears at the eighteenth amino acid (Lys), implying that minor volume substituents might be adverse for the activity at this site. For instance, peptides 23 possessing Phe at this position exhibits higher activity compared to peptide 24 with Glu. Around the twentieth amino acid (Leu), a large green contour map is situated, indicating that large groups are beneficial for the activity. This might be the reason why the activity of peptide 30 (Arg) is higher than that of peptide 29 (Thr).

The electrostatic contour maps of CoMFA model are shown in Fig. 9B, the blue contour maps mean that electropositive groups are beneficial for improving activity, while the red contour maps mean the electronegative substituents are favored. There are several blue contour maps over the first amino acid (Lys), suggesting that adding positive charged groups at this position will improve the activity. This may explain why the activity of peptide 27 with Arg is greater than that of peptide 26 with Ser. A blue contour map is located near the second amino acid (Ile), illustrating that positively charged groups at this position can enhance the activity. For the most active peptide 22, the amino acid is Ile, thus, modifications can be made at this site according to the electrostatic property. A large blue contour map covering the seventh amino acid (Leu) indicates that electropositive groups in this region generally get good activity. This is in good agreement with the experimental data: 27 (Thr) > 28 (Gln). There is a blue contour map located at the eighth amino acid, indicating that peptides with electropositive groups are beneficial for the activity. An irregular blue contour map is near to the ninth amino acid, suggesting that positive electrostatic substituents here are important for increasing the activity, as observed from peptide 27 (Arg) > peptide 28 (Leu). A blue contour appearing over the position of tenth amino acid (Leu) illustrates that this region is suitable for improving the electropositivity. Thus, the activity of peptide 27 with Arg is significantly improved compared with peptide 28 (Gly). There is a large red contour map around the thirteenth amino acid (Ala), suggesting that the introduction of negative moieties into this position would be beneficial to the activity. Therefore, modifications can be made at this site to enhance the activity. Around the fourteenth amino acid (Lys), a blue contour map is located, indicating that the presence of electropositive groups are suitable. This is well illustrated by the order of activity for these peptides: peptide 23 (Arg) > peptide 24 (Ala). A moderate red contour is found close to the seventeenth amino acid (Leu), indicating negative charged groups are favored. This trend can be reflected by the activities of peptides 23 and 24: peptide 23 (Asp) > peptide 24 (Gly). In addition, the eighteenth amino acid (Lys) is surrounded by a blue contour map, suggesting that electropositive potential is preferred, this can be verified by the fact that the Leu (peptide 20) confers an activity disadvantage over the corresponding Lys (peptide 19). A red contour map is observed close to the twentieth amino acid (Leu), indicating that electronegative groups at this position are favorable to the activity, therefore, structural modifications can be made here based on the electrostatic information.

**Fig. 10:**
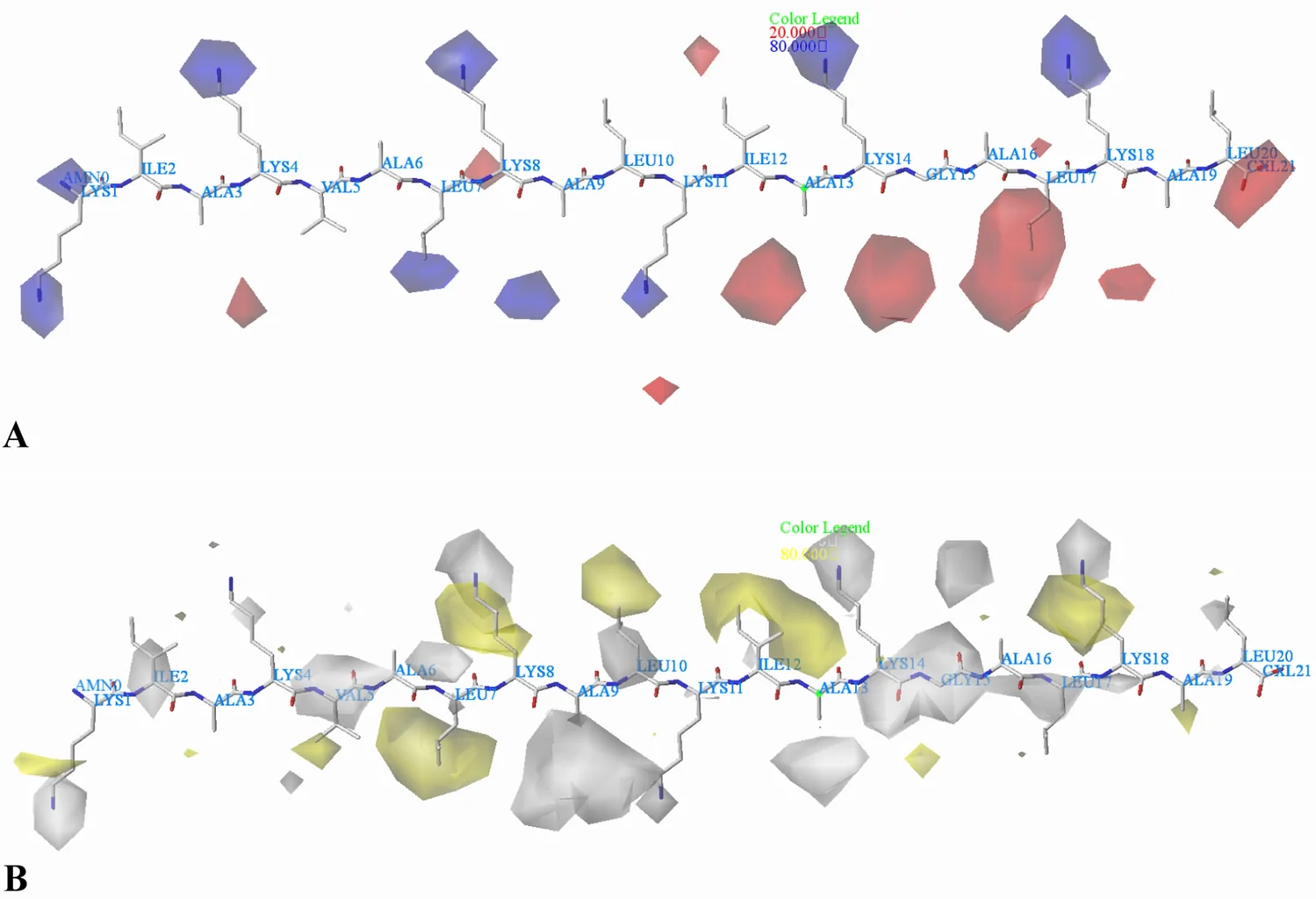
CoMSIA StDev*Coeff contour plots for *Escherichia coli* in combination of peptide 22. **A** The electrostatic contour map, where the blue and red contours represent 80% and 20% level contributions, respectively. **B** The hydrophobic contour map, where the yellow and white contours represent 80% and 20% level contributions, respectively

As shown in Fig. 10A, it can be observed that the electrostatic contour maps of the CoMSIA model are highly similar to those of the CoMFA model. The main difference is that a blue contour map is observed at the fourth amino acid (Lys), indicating that positively charged groups are preferred, for example, the activity of peptide 27 (Arg) is higher than peptide 28 (Thr). Furthermore, another blue contour is situated around the eleventh amino acid (Lys), suggesting that the activity can be improved if the group is positively charged and that the activity of peptide 13 (Lys) is superior to that of peptide 14 (Ser).

The hydrophobic contour plots of the CoMSIA model are presented in Fig. 10B, the yellow and white contour maps show the favorable and unfavorable hydrophobic interactions, respectively. A small white plot is found around the first amino acid (Lys) indicating the favorable region for the presence of hydrophilic groups. A white contour plot located on the second amino acid (Ile) indicates that hydrophilic groups favor the activity. This can be explained by the fact that peptide 30 having Arg at this position, favors the activity while peptide 31 having hydrophobic amino acid Val. A white contour at the fourth amino acid (Lys) demonstrates the favorable effect of hydrophilic groups in increasing the activity, which can explain why peptide 30 with hydrophilic amino acid Arg shows higher activity than peptide 31 with hydrophobic residue Phe. Besides, a yellow contour located at the fifth amino acid (Val) suggests that the hydrophobic groups would enhance the activity. The activity order 1 (Arg) > 2 (Ala) is a corresponding example. There is a yellow contour over the seventh amino acid (Leu), suggesting that adding hydrophobic groups at this position will increase the activity. This may explain why the activity of peptide 17 with Leu is greater than that of peptide 15 with an amino acid Arg. Around the eighth (Lys) amino acid, a yellow and a white contour map are distributed at the same time, indicating that the substituents at this location need to be chosen carefully. A white contour is located near the ninth amino acid (Ala), indicating that peptides with hydrophilic groups at this position might possess better activity. For instance, the activity of 27 (Arg) is higher than 28 (Leu). There is a white contour at the substituent of the tenth amino acid, and a yellow contour at the terminal of the residue, suggesting that it is necessary to select the substituent carefully according to the hydrophobic characteristics. A small white contour is located at the eleventh amino acid (Lys), suggesting that hydrophilic groups are important for the activity. For example, peptide 27 (Arg) exhibits higher activity than peptide 28 (Leu). A yellow contour seen near the twelfth amino acid (Ile) indicates that hydrophobic substitution is favored in this region, this might be the reason for higher activity of peptide 23 (Val) than peptide 24 (Gly). A white contour near the thirteenth amino acid (Ala) suggests that hydrophilic substitution in this region could increase the activity. A white contour near the fourteenth amino acid (Lys) implies that hydrophilic substitution in this area could increase the activity of the peptide. In the case of peptides 23 and 24, peptide 23, consisting of residue Arg is more potent than peptide 24 (Ala). A white contour map at the fifteenth amino acid (Gly) indicates that hydrophilic substitution is found to be favorable for enhanced activity. For example, peptide 22 with hydrophilic group Gly is more active than peptide 21 with hydrophobic Val. At the eighteenth amino acid (Lys), a yellow and a white contour map are situated. Furthermore, we can see that there is a small yellow contour that appeared near the nineteenth amino acid (Ala), which indicate that adding hydrophobic substituents might increase activity. There are several white contours near the twentieth amino acid (Leu), which could conclude that hydrophilic groups would increase activities, such as peptide 23 (Arg) has better activity than peptide 24 (Met).

#### Contour maps for *Staphylococcus aureus*

**Fig. 11:**
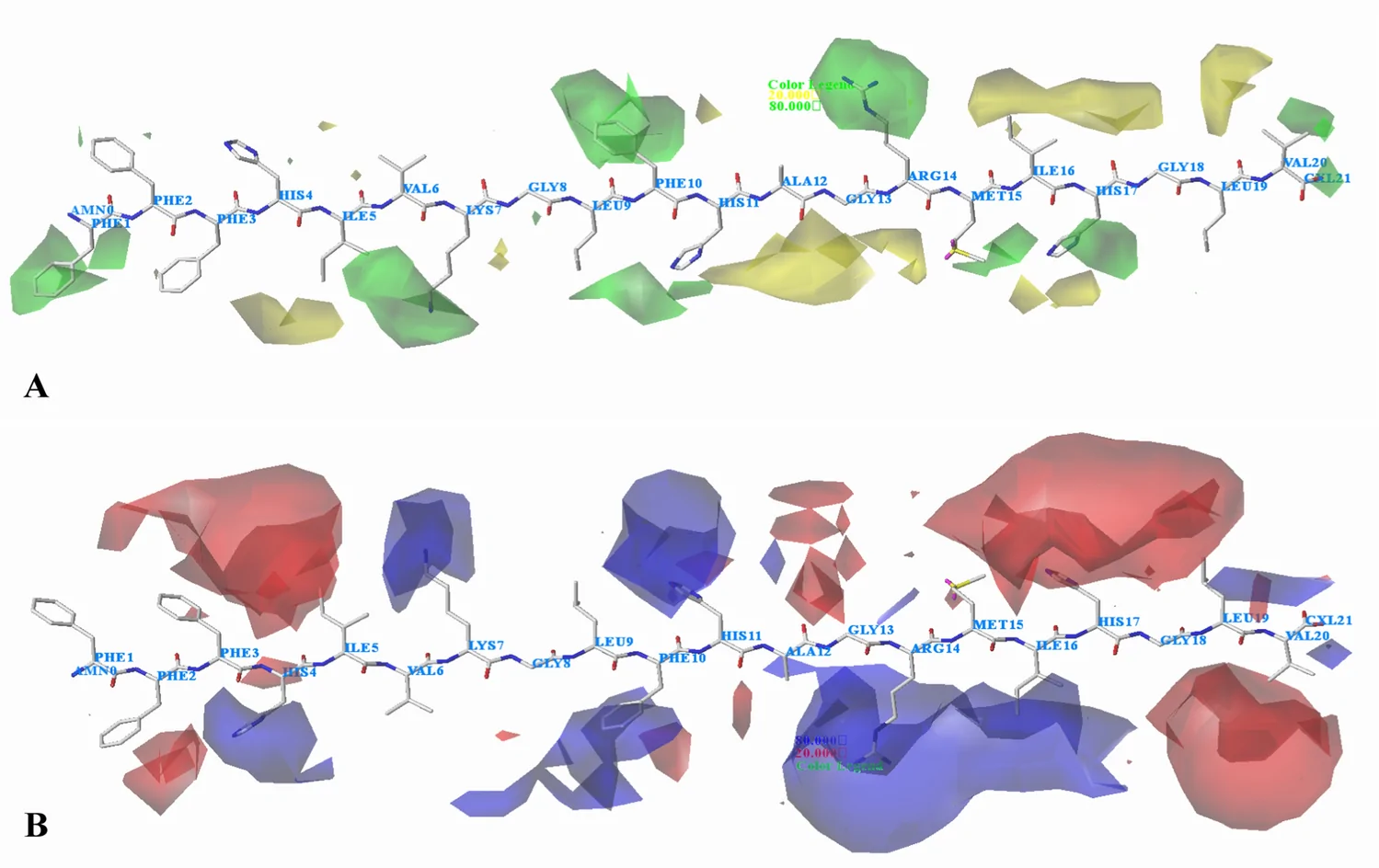
CoMFA StDev*Coeff contour plots for *Staphylococcus aureus* in combination of peptide 13. **A** The steric contour map, where the green and yellow contours represent 80% and 20% level contributions, respectively. **B** The electrostatic contour map, where the blue and red contours represent 80% and 20% level contributions, respectively

The CoMFA steric contour map for *Staphylococcus aureus* is shown in Fig. 11A. A green contour located at the first amino acid (Phe) indicates that bulkier substituent is favored at the position. Two minor green contours are shown near the second amino acid (Phe), suggesting that  large substituents would be preferable on this site, which agrees well with the experimental results. For example, the activity follows the order: peptide 21 (Ile) > peptide 20 (Leu), peptide 40 (Phe) > peptide 41 (Ile). In addition, there is a small yellow contour near the third amino acid (Phe), showing that the existence of a minor group may improve the activity, as observed in peptide 18 (Gly) > peptide 17 (Ala). The fourth amino acid Ile is observed in the region of green contour, which means larger groups in this region might increase the activity. Different activities of peptides 5 (Lys) and 6 (Arg) (peptide 5 < peptide 6) are probably caused by the green contour. A yellow polyhedron contour around the fifth amino acid (Ile) indicates that small groups are favorable to the activity. For instance, the order of activity is as follows: peptide 2 (Ala) > peptide 1 (Arg). A large green contour map can be seen around the seventh amino acid (Lys), indicating that steric property in this region might increase the activity. For instance, the activity of peptide 25 (Lys) is relatively higher than that of 26 (Ile). The ninth amino acid (Leu) is encompassed by a green contour map, which indicate that bulky substitution to this position could enhance the activity. For example, peptide 1, bearing Phe shows higher activity than peptide 3 (Ala). A large green contour is situated around the tenth amino acid (Phe), suggesting that bulky groups are favored. Peptide 21 with Leu shows higher potency than peptide 20 with Gly. Two green contour maps are located near the eleventh amino acid (His), suggesting that bulky groups at this position would be favorable to the activity, whereas several yellow regions near the thirteenth amino acid (Gly) suggest that bulky groups at this position would decrease the activity. There is a green contour covering the fourteenth amino acid (Arg), illustrating that bulky groups at this area can improve the activity. It is possible to explain why the activity of peptide 15 (Arg) is higher than that of peptide 14 (Thr). In addition, we can see a green contour that covers the fifteenth amino acid (Met), showing that the substitution in this position is favorable for increasing the activity, which can explain that peptide 38 with Val substitution is more active than peptide 39 with Gly. A large and a small yellow contours cover the sixteenth amino acid, which suggests that minor group selection is required in this region. For example, peptide 2 introduces Ala in this area and its activity improved compared with peptide 1 (Lys). There is a green contour at the seventeenth amino acid, illustrating large substituent is beneficial to the activity, such as the activity of peptide 30 (Lys) is greater than peptide 31 (Ala). Several green contour maps appear near the twentieth amino acid (Val), indicating that the bulky volume is conducive to the activity (peptide 25 with Lys > peptide 26 with Gly).

Figure 11B depicts the electrostatic field contour maps of CoMSIA model. Red contours shown near the second amino acid (Phe) and the third amino acid (Phe) indicate that the electronegative groups are favorable, therefore, modifications can be made at these positions according to the electrostatic property. A blue contour is observed surrounding the fourth amino acid (His), which suggest that electropositive groups would increase the activity. It can be seen in case of peptides 13 and 40: peptide 13 (His) > peptide 40 (Pro). A red contour at the fifth amino acid (Ile) indicates that negatively charged groups would enhance the activity. Thus, peptide 2 with Ala exhibits increased activity when compared with peptide 1 (Arg). A blue contour surrounding the seventh amino acid (Lys) indicates that peptides with electropositive substitution can possess better activity, for example, the activity of peptide 25 (Lys) is higher than that of peptide 26 (Ile). Several blue contours located at the tenth amino acid (Phe) show the importance of electropositive atoms in imparting better activity. For a consideration, the activity rank for peptides are 25 (Lys) > 26 (Ala). A blue contour near the eleventh amino acid (His) expresses that the electropositive groups are preferred here. Some large blue contours near the fourteenth amino acid (Arg) indicate that electropositive groups are favored. This is consistent with the experimental results that peptide 15 with Arg is more active than peptide 14 (Thr). A red contour near the fifteenth amino acid (Met) suggests that  electronegative substituent would increase the activity. This is in good correlation with the experimental activities (peptide 2 with Glu > peptide 4 with Gln). A blue contour near the sixteenth amino acid (Ile) indicates the  electropositive group is beneficial to the activity. This can be explained by comparing the order of activities in these series: peptide 15 (Arg) > peptide 14 (Thr). There is a blue contour over the nineteenth amino acid (Leu), suggesting that adding positive charged groups at this position will increase the activity, this may explain why the activity of peptide 18 with Lys is greater than that of peptide 17 with Ala. Furthermore, several red contours at the seventeenth amino acid (His) and a red contour at the twentieth amino acid (Val) illustrate that electronegative groups are favored at these locations, thus, modifications can be made.

**Fig. 12:**
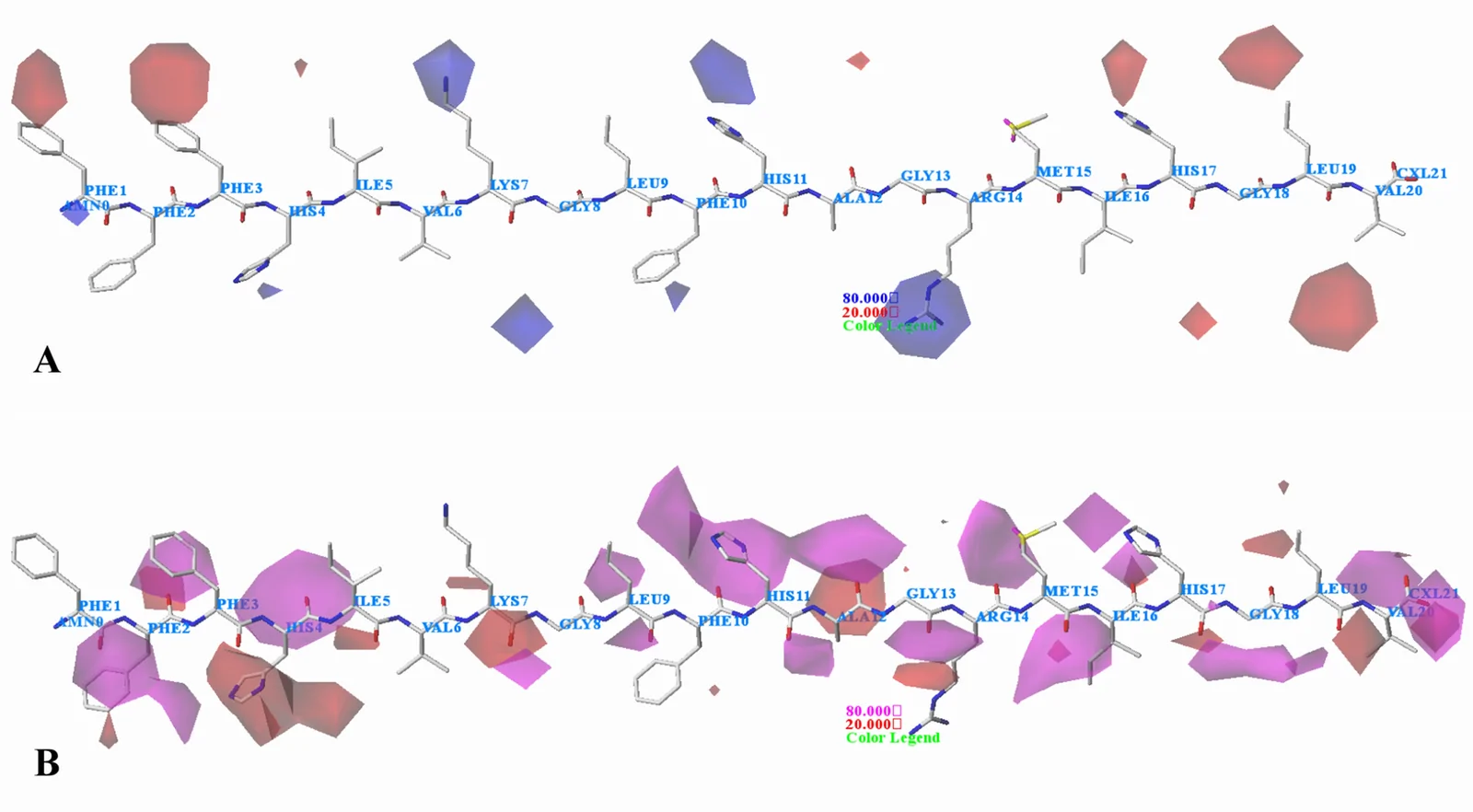
CoMSIA StDev*Coeff contour plots for *Staphylococcus aureus* in combination of peptide 13. **A** The electrostatic contour map, where the blue and red contours represent 80% and 20% level contributions, respectively. **B** The hydrogen bond acceptor contour map, where the magenta and red contours represent 80% and 20% level contributions, respectively

The CoMSIA electrostatic contour plots are shown in Fig. 12A and are similar to those obtained in CoMFA analysis. However, differences are also existed, the first difference is that a red contour map is positioned at the first amino acid, suggesting that negative groups are favored at this position. In addition, there is a red contour located at the nineteenth amino acid, indicating that careful selection should made for this place.

In hydrogen bond acceptor field (Fig. 12B), the magenta and red contours indicate favorable and unfavorable hydrogen bond acceptor groups. Magenta contours are found near the second amino acid (Phe) and the third amino acid (Phe), indicating that hydrogen bond acceptor groups are favorable for the activity. Around the fourth amino acid (His), a red contour map is situated, suggesting that hydrogen bond acceptor groups are disfavored, therefore the higher activity of peptide 13 (His) over peptide 40 (Pro), expected like as experimental data. The magenta contour map around the ninth amino acid (Leu) expresses that the presence of hydrogen bond acceptor group would increase the activity. A magenta contour covering the eleventh amino acid (His) indicates that hydrogen bond acceptor group in this region generally gets better activity. This is in good agreement with the experimental data: peptide 13 (His) > peptide 40 (Lys). There is a magenta contour located in the position of the twelfth amino acid (Ala), illustrating that peptides with hydrogen bond acceptor groups are good for the activity. A magenta contour is seen near the fifteenth amino acid (Met), indicating that hydrogen bond acceptor groups are desired in this region. It is noteworthy that the activity of peptide 13 with Met is higher than peptide 40 with Ile. Another magenta contour is located near the sixteenth amino acid (Ile), suggesting that hydrogen bond acceptor group in this area is needed. For example, peptide 21 with His is more active than peptide 20 with Leu. At the seventeenth amino acid, a magenta contour is situated, further reflecting that more potent peptides contain hydrogen bond acceptor groups in this region. For instance, peptide 7 that contains Pro has better activity than peptide 5 with Ser. The nineteenth amino acid Leu is surrounded by two red contours, illustrating that hydrogen bond donor groups are favored at this position. This may explain why peptide 18 with Lys shows increased activity than peptide 17 with Ala. A red contour is found close to the twentieth amino acid (Val), indicating hydrogen bond donor groups are favored. This is well illustrated by the order of activity for these peptides: 25 (Lys) > 26 (Gly).

### Applicability domain

For *Escherichia coli*, the superior models for 2D-QSAR and 3D-QSAR are SVR and CoMFA, respectively. Additionally, it is the same for the series of *Staphylococcus aureus*. Therefore, applicability domain was only conducted on these models, the results show that no outliers were found for the training set and test set in all models, indicating that the developed 2D/3D-QSAR models are reliable and can be used for predicting the activities of novel peptides.

### Designing potent peptides

Based on the constructed 2D-QSAR and 3D-QSAR models, some new peptides targeting *Escherichia coli* and *Staphylococcus aureus* have been designed to improve the activity, as shown in Table 10. All these peptides have been minimized and aligned to the original dataset, then the activities were predicted. In addition, the toxicity of the designed peptides were predicted by the online website ToxinPred3 (https://webs.iiitd.edu.in/raghava/toxinpred3), which can be used to predict the toxicity of peptides, in addition to designing the least toxic peptides and discovering toxic regions in proteins, and the results are listed in Table S3, illustrating that none of the six designed peptides is toxic. Finally, we find that these peptides would be ideal as candidates for experimental synthesis.

**Table 10:** Structures of newly designed peptides based on developed models

No	Sequence	Predicted pIC_50_(μM) for *Escherichia coli*		Predicted pIC_50_(μM) for* Staphylococcus aureus*	
		CoMFA	SVR	CoMFA	SVR
D1	KLAKAALRARRIDWGDLLRL	6.244	6.245	5.913	5.916
D2	KIAKVALRARRIDRGDLLRL	6.237	6.240	5.913	5.915
D3	KLAKAALRARRIDWGDFFRL	6.233	6.235	5.904	5.910
D4	KIAKAAIRARRIDWGDFFRL	6.238	6.237	5.915	5.917
D5	KLAKAALRARKIDHGDLLRL	6.241	6.242	5.914	5.916
D6	KLFRAALRARKIDWGDLLRL	6.242	6.240	5.913	5.914

### Prediction of designed peptides transmembrane activity and DNA interaction activity

The transmembrane property of the designed peptides was predicted using DeepTMHMM (https://dtu.biolib.com/DeepTMHMM) (Table S4), the results showed that all the designed peptides had no transmembrane region, thus the cell membrane was not the main target, there may be intracellular sites of action.

**Fig. 13:**
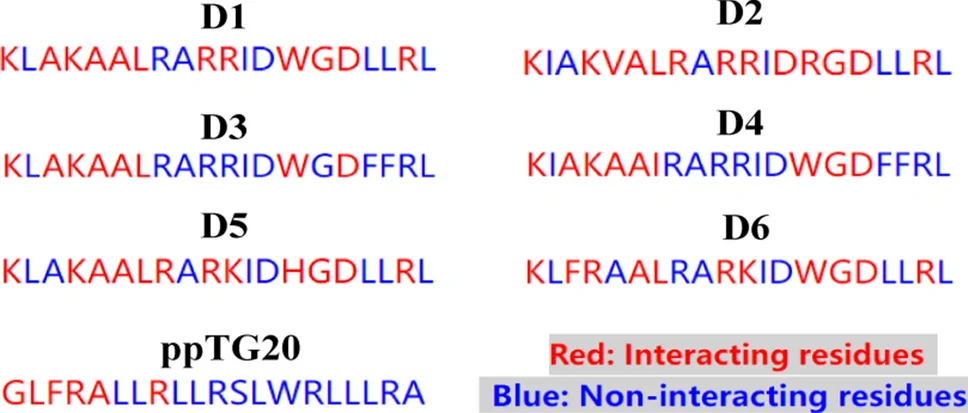
The DNA-binding positions. The binding residues were labeled with red; the non-binding residues were labeled with blue

The results of the DNA-binding activity analysis for the designed peptides are shown in Fig. 13, and the results show that all the designed peptides have higher number of DNA-binding sites than the parent peptide PPTG20. In addition, D2 has the highest number of binding sites. The binding sites of all the designed peptides to DNA were dispersed in the sequence, and the binding sites were relatively diversified, It contains some amino acid residues with better nucleic acid-protein binding preference, including some amino acid residues with better nucleic acid-protein binding preference, such as amino acid R, K, D, W, further indicating that the binding probability of the designed antibacterial peptides to DNA is higher. Therefore, further in-depth in vitro experimental studies can be conducted.

## Conclusion

In this work, different peptides targeting *Escherichia coli* and *Staphylococcus aureus* were studied using 2D-QSAR and 3D-QSAR models to explore the structure–activity relationship. The constructed QSAR models especially CoMFA and SVR models possessed excellent predictive power. Furthermore, the CoMFA contour maps along with the information of molecular descriptors offer critical information affecting the activity of these peptides and explicit indications for the design of better peptides.

The statistical results of the derived models were used to design novel peptides, and some new peptides were produced by changing structure features of the most potent peptide 22 (*Escherichia coli*), peptide 13 (*Staphylococcus aureus*) and the penetrating peptide ppTG20. The activities of the newly designed peptides were also predicted and were found to be more potent than the corresponding parent peptides. Therefore, the strategy implemented in the present work could be useful for designing more potent peptides.

## Supplementary Information

Below is the link to the electronic supplementary material.

Supplementary file1 (DOCX 41 KB)

## Data Availability

Data sharing is not applicable to this article as the corresponding data have been shown in the article, and no new data were created in this study.
