# Supplementary material for: Hybrid 2D/3D-quantitative structure–activity relationship studies on the bioactivities and molecular mechanism of antibacterial peptides
Source: Amino Acids. 2024 Feb 15;56(1):16. doi: 10.1007/s00726-024-03381-x (PMC10869384; doi:10.1007/s00726-024-03381-x)
Supplement: Supplementary file 1 — Supplementary file1 (DOCX 41 KB) [file 726_2024_3381_MOESM1_ESM.docx]

**Hybrid 2D/3D-quantitative structure-activity relationship studies on the bioactivities and molecular mechanism of antibacterial peptides**

**Qingguo Yan^a1^, Fangfang Wang^a1^**^*^, **Bo Zhou^b^, Xiangna Lin^a^**^*^

^a^ School of Life Science, Linyi University, Linyi, 276000, China;

^b^ State Key Laboratory of Functions and Applications of Medicinal Plants, College of Basic Medical, Guizhou Medical University, Guizhou, 550004, China.

* Corresponding author

E-mail: yu100288@163.com

**Table S1** Summary of QSAR results for antibacterial peptides targeted on *E.coli* based on template ligand-based alignment.

|  | | CoMFA | | CoMSIA | | | | | | | | | | | | | | | | | | |
| --- | --- | --- | --- | --- | --- | --- | --- | --- | --- | --- | --- | --- | --- | --- | --- | --- | --- | --- | --- | --- | --- | --- |
|  |  | SE | | S | | | E | | H | | | | D | A | | | SE | | | | SH | SD |
| R^2^_cv_ | | 0.537 | | 0.424 | | | 0.498 | | 0.546 | | | | 0.325 | 0.355 | | | 0.520 | | | | 0.536 | 0.330 |
| R^2^_ncv_ | | 0.822 | | 0.997 | | | 0.987 | | 0.976 | | | | 0.565 | 0.676 | | | 0.992 | | | | 0.985 | 0.776 |
| SEE | | 0.232 | | 0.040 | | | 0.069 | | 0.093 | | | | 0.355 | 0.314 | | | 0.058 | | | | 0.076 | 0.261 |
| F | | 46.240 | | 371.723 | | | 207.114 | | 136.365 | | | | 27.245 | 20.833 | | | 253.945 | | | | 172.205 | 34.613 |
| R^2^_pred_ | | 0.5001 | | 0.0347 | | | 0.2743 | | 0.08084 | | | | 0.1625 | 0.1644 | | | 0.2965 | | | | 0.0892 | 0.4093 |
| SEP | | 0.375 | | 0.540 | | | 0.436 | | 0.402 | | | | 0.442 | 0.442 | | | 0.441 | | | | 0.420 | 0.451 |
| N_C_ | | 2 | | 10 | | | 6 | | 5 | | | | 1 | 2 | | | 7 | | | | 6 | 2 |
| **Field contribution** | | | | | | | | | | | | | | | | | | | | | | |
| S | | 0.451 | | 1.000 | | | - | | - | | | | - | - | | | 0.334 | | | | 0.329 | 0.380 |
| E | | 0.549 | | - | | | 1.000 | | - | | | | - | - | | | 0.666 | | | | - | - |
| H | | - | | - | | | - | | 1.000 | | | | - | - | | | - | | | | 0.671 | - |
| D | | - | | - | | | - | | - | | | | 1.000 | - | | | - | | | | - | 0.620 |
| A | | - | | - | | | - | | - | | | | - | 1.000 | | | - | | | | - | - |
| CoMSIA | | | | | | | | | | | | | | | | | | | | | | |
|  | | SA | | EH | | | ED | EA | | | | HD | | HA | | | | DA | | | SEH | SED |
| R^2^_cv_ | | 0.434 | | 0.512 | | | 0.384 | 0.465 | | | | 0.390 | | 0.504 | | | | 0.315 | | | 0.522 | 0.407 |
| R^2^_ncv_ | | 0.993 | | 0.980 | | | 0.983 | 0.979 | | | | 0.967 | | 0.985 | | | | 0.515 | | | 0.980 | 0.987 |
| SEE | | 0.055 | | 0.085 | | | 0.080 | 0.087 | | | | 0.105 | | 0.076 | | | | 0.374 | | | 0.084 | 0.069 |
| F | | 251.431 | | 166.016 | | | 155.754 | 156.769 | | | | 133.803 | | 170.382 | | | | 22.336 | | | 169.963 | 208.415 |
| R^2^_pred_ | | 0.0481 | | 0.5675 | | | 0.2981 | 0.3864 | | | | 0.1999 | | 0.0657 | | | | 0.2219 | | | 0.3835 | 0.3186 |
| SEP | | 0.495 | | 0.418 | | | 0.484 | 0.465 | | | | 0.453 | | 0.434 | | | | 0.445 | | | 0.413 | 0.474 |
| N_C_ | | 8 | | 5 | | | 6 | 5 | | | | 4 | | 6 | | | | 1 | | | 5 | 6 |
| **Field contribution** | | | | | | | | | | | | | | | | | | | | | | |
| S | 0.492 | | | | - | | - | | | - | | | - | - | | | - | | | | 0.201 | 0.216 |
| E | - | | | | 0.522 | | 0.550 | | | 0.668 | | | - | - | | | - | | | | 0.418 | 0.427 |
| H | - | | | | 0.478 | | - | | | - | | | 0.526 | 0.652 | | | - | | | | 0.381 | - |
| D | - | | | | - | | 0.450 | | | - | | | 0.474 | - | | | 0.527 | | | | - | 0.357 |
| A | 0.508 | | | | - | | - | | | 0.332 | | | - | 0.348 | | | 0.473 | | | | - | - |
| CoMSIA | | | | | | | | | | | | | | | | | | | | | | |
|  | SEA | | | | SHD | | SHA | | | SDA | | | EHD | | EHA | | | | EDA | | HDA | SEHD |
| R^2^_cv_ | 0.493 | | | | 0.408 | | 0.505 | | | 0.340 | | | 0.431 | | 0.501 | | | | 0.376 | | 0.384 | 0.439 |
| R^2^_ncv_ | 0.985 | | | | 0.979 | | 0.986 | | | 0.751 | | | 0.990 | | 0.979 | | | | 0.987 | | 0.978 | 0.980 |
| SEE | 0.076 | | | | 0.087 | | 0.074 | | | 0.275 | | | 0.062 | | 0.086 | | | | 0.069 | | 0.089 | 0.085 |
| F | 173.215 | | | | 158.795 | | 181.419 | | | 30.228 | | | 256.573 | | 161.998 | | | | 209.32 | | 150.881 | 166.103 |
| R^2^_pred_ | 0.3496 | | | | 0.1458 | | 0.0697 | | | 0.3168 | | | 0.3222 | | 0.2932 | | | | 0.2670 | | 0.1127 | 0.3465 |
| SEP | 0.439 | | | | 0.460 | | 0.433 | | | 0.448 | | | 0.465 | | 0.422 | | | | 0.487 | | 0.469 | 0.476 |
| N_C_ | 6 | | | | 5 | | 6 | | | 2 | | | 6 | | 5 | | | | 6 | | 4 | 5 |
| **Field contribution** | | | | | | | | | | | | | | | | | | | | | | |
| S | 0.240 | | | | 0.215 | | 0.248 | | | 0.266 | | | - | | - | | | | - | | - | 0.152 |
| E | 0.511 | | | | - | | - | | | - | | | 0.360 | | 0.415 | | | | 0.425 | | - | 0.307 |
| H | - | | | | 0.410 | | 0.493 | | | - | | | 0.341 | | 0.377 | | | | - | | 0.408 | 0.280 |
| D | - | | | | 0.375 | | - | | | 0.443 | | | 0.299 | | - | | | | 0.349 | | 0.367 | 0.261 |
| A | 0.249 | | | | - | | 0.258 | | | 0.291 | | | - | | 0.207 | | | | 0.226 | | 0.225 | - |
| CoMSIA | | | | | | | | | | | | | | | | | | | | | | |
|  | SEHA | | | | | SEDA | | | | | SHDA | | | | | EHDA | | | | SEHDA | | |
| R^2^_cv_ | 0.510 | | | | | 0.396 | | | | | 0.402 | | | | | 0.421 | | | | 0.434 | | |
| R^2^_ncv_ | 0.986 | | | | | 0.990 | | | | | 0.985 | | | | | 0.979 | | | | 0.992 | | |
| SEE | 0.074 | | | | | 0.062 | | | | | 0.075 | | | | | 0.087 | | | | 0.058 | | |
| F | 183.800 | | | | | 223.416 | | | | | 177.935 | | | | | 157.977 | | | | 252.805 | | |
| R^2^_pred_ | 0.2736 | | | | | 0.2873 | | | | | 0.1203 | | | | | 0.2882 | | | | 0.2599 | | |
| SEP | 0.431 | | | | | 0.494 | | | | | 0.476 | | | | | 0.455 | | | | 0.479 | | |
| N_C_ | 6 | | | | | 7 | | | | | 6 | | | | | 5 | | | | 7 | | |
| **Field contribution** | | | | | | | | | | | | | | | | | | | | | | |
| S | | | 0.166 | | | 0.179 | | | | | 0.176 | | | | | - | | | | 0.135 | | |
| E | | | 0.346 | | | 0.348 | | | | | - | | | | | 0.307 | | | | 0.262 | | |
| H | | | 0.318 | | | - | | | | | 0.338 | | | | | 0.279 | | | | 0.247 | | |
| D | | | - | | | 0.292 | | | | | 0.304 | | | | | 0.257 | | | | 0.222 | | |
| A | | | 0.170 | | | 0.182 | | | | | 0.182 | | | | | 0.157 | | | | 0.135 | | |

**Table S2** Summary of QSAR results for antibacterial peptides targeted on *Staphylococcus aureus* based on template ligand-based alignment.

|  | | CoMFA | | CoMSIA | | | | | | | | | | | | | | | | | | |
| --- | --- | --- | --- | --- | --- | --- | --- | --- | --- | --- | --- | --- | --- | --- | --- | --- | --- | --- | --- | --- | --- | --- |
|  |  | SE | | S | | | E | | H | | | D | | A | | | SE | | | | SH | SD |
| R^2^_cv_ | | 0.607 | | 0.526 | | | 0.588 | | 0.187 | | | 0.554 | | 0.207 | | | 0.585 | | | | 0.399 | 0.555 |
| R^2^_ncv_ | | 0.970 | | 0.963 | | | 0.965 | | 0.995 | | | 0.984 | | 0.893 | | | 0.977 | | | | 0.996 | 0.987 |
| SEE | | 0.111 | | 0.122 | | | 0.119 | | 0.050 | | | 0.086 | | 0.208 | | | 0.098 | | | | 0.046 | 0.078 |
| F | | 153.531 | | 125.681 | | | 132.29 | | 389.51 | | | 159.951 | | 40.091 | | | 166.295 | | | | 456.197 | 195.32 |
| R^2^_pred_ | | 0.5883 | | 0.0847 | | | 0.3779 | | 0.0001 | | | 0.1050 | | 0.3016 | | | 0.5083 | | | | 0.0030 | 0.1022 |
| SEP | | 0.399 | | 0.438 | | | 0.408 | | 0.645 | | | 0.454 | | 0.567 | | | 0.418 | | | | 0.554 | 0.454 |
| N_C_ | | 5 | | 5 | | | 5 | | 10 | | | 8 | | 5 | | | 6 | | | | 10 | 8 |
| **Field contribution** | | | | | | | | | | | | | | | | | | | | | | |
| S | | 0.408 | | 1.000 | | | - | | - | | | | - | - | | | 0.287 | | | | 0.339 | 0.308 |
| E | | 0.592 | | - | | | 1.000 | | - | | | | - | - | | | 0.713 | | | | - | - |
| H | | - | | - | | | - | | 1.000 | | | | - | - | | | - | | | | 0.661 | - |
| D | | - | | - | | | - | | - | | | | 1.000 | - | | | - | | | | - | 0.692 |
| A | | - | | - | | | - | | - | | | | - | 1.000 | | | - | | | | - | - |
| CoMSIA | | | | | | | | | | | | | | | | | | | | | | |
|  | | SA | | EH | | | ED | EA | | | | HD | | HA | | | | DA | | | SEH | SED |
| R^2^_cv_ | | 0.472 | | 0.463 | | | 0.567 | 0.556 | | | | 0.407 | | 0.243 | | | | 0.525 | | | 0.490 | 0.569 |
| R^2^_ncv_ | | 0.984 | | 0.978 | | | 0.978 | 0.969 | | | | 0.992 | | 0.980 | | | | 0.982 | | | 0.975 | 0.982 |
| SEE | | 0.085 | | 0.097 | | | 0.096 | 0.112 | | | | 0.064 | | 0.094 | | | | 0.090 | | | 0.100 | 0.088 |
| F | | 166.032 | | 169.109 | | | 170.245 | 151.151 | | | | 233.391 | | 155.399 | | | | 146.048 | | | 191.04 | 175.064 |
| R^2^_pred_ | | 0.0018 | | 0.2282 | | | 0.5018 | 0.5371 | | | | 0.0331 | | 0.0445 | | | | 0.0145 | | | 0.2547 | 0.1983 |
| SEP | | 0.494 | | 0.476 | | | 0.427 | 0.424 | | | | 0.551 | | 0.578 | | | | 0.469 | | | 0.454 | 0.474 |
| N_C_ | | 8 | | 6 | | | 6 | 5 | | | | 10 | | 7 | | | | 8 | | | 5 | 7 |
| **Field contribution** | | | | | | | | | | | | | | | | | | | | | | |
| S | 0.529 | | | | - | | - | | | - | | | - | - | | | - | | | | 0.182 | 0.175 |
| E | - | | | | 0.571 | | 0.530 | | | 0.711 | | | - | - | | | - | | | | 0.468 | 0.436 |
| H | - | | | | 0.429 | | - | | | - | | | 0.459 | 0.663 | | | - | | | | 0.350 | - |
| D | - | | | | - | | 0.470 | | | - | | | 0.541 | - | | | 0.695 | | | | - | 0.389 |
| A | 0.471 | | | | - | | - | | | 0.289 | | | - | 0.337 | | | 0.305 | | | | - | - |
| CoMSIA | | | | | | | | | | | | | | | | | | | | | | |
|  | SEA | | | | SHD | | SHA | | | SDA | | | EHD | | EHA | | | | EDA | | HDA | SEHD |
| R^2^_cv_ | 0.561 | | | | 0.456 | | 0.383 | | | 0.538 | | | 0.494 | | 0.456 | | | | 0.552 | | 0.410 | 0.510 |
| R^2^_ncv_ | 0.975 | | | | 0.989 | | 0.984 | | | 0.985 | | | 0.979 | | 0.976 | | | | 0.977 | | 0.986 | 0.980 |
| SEE | 0.102 | | | | 0.071 | | 0.085 | | | 0.082 | | | 0.094 | | 0.102 | | | | 0.099 | | 0.079 | 0.092 |
| F | 151.671 | | | | 237.133 | | 188.016 | | | 176.326 | | | 180.684 | | 152.858 | | | | 160.79 | | 190.359 | 185.45 |
| R^2^_pred_ | 0.1865 | | | | 0.0516 | | 0.0127 | | | 0.0195 | | | 0.1978 | | 0.1227 | | | | 0.1649 | | 0.0088 | 0.1873 |
| SEP | 0.431 | | | | 0.501 | | 0.522 | | | 0.462 | | | 0.462 | | 0.479 | | | | 0.435 | | 0.522 | 0.455 |
| N_C_ | 6 | | | | 8 | | 7 | | | 8 | | | 6 | | 6 | | | | 6 | | 8 | 6 |
| **Field contribution** | | | | | | | | | | | | | | | | | | | | | | |
| S | 0.215 | | | | 0.192 | | 0.255 | | | 0.237 | | | - | | - | | | | - | | - | 0.128 |
| E | 0.559 | | | | - | | - | | | - | | | 0.378 | | 0.465 | | | | 0.438 | | - | 0.330 |
| H | - | | | | 0.370 | | 0.493 | | | - | | | 0.288 | | 0.346 | | | | - | | 0.370 | 0.251 |
| D | - | | | | 0.439 | | - | | | 0.529 | | | 0.334 | | - | | | | 0.380 | | 0.439 | 0.290 |
| A | 0.226 | | | | - | | 0.252 | | | 0.233 | | | - | | 0.190 | | | | 0.181 | | 0.190 | - |
| CoMSIA | | | | | | | | | | | | | | | | | | | | | | |
|  | SEHA | | | | | SEDA | | | | | SHDA | | | | | EHDA | | | | SEHDA | | |
| R^2^_cv_ | 0.484 | | | | | 0.561 | | | | | 0.454 | | | | | 0.487 | | | | 0.500 | | |
| R^2^_ncv_ | 0.977 | | | | | 0.978 | | | | | 0.988 | | | | | 0.978 | | | | 0.979 | | |
| SEE | 0.099 | | | | | 0.097 | | | | | 0.075 | | | | | 0.097 | | | | 0.095 | | |
| F | 161.808 | | | | | 168.116 | | | | | 214.126 | | | | | 167.556 | | | | 175.754 | | |
| R^2^_pred_ | 0.1137 | | | | | 0.1614 | | | | | 0.0118 | | | | | 0.1189 | | | | 0.1095 | | |
| SEP | 0.467 | | | | | 0.431 | | | | | 0.502 | | | | | 0.465 | | | | 0.479 | | |
| N_C_ | 6 | | | | | 6 | | | | | 8 | | | | | 6 | | | | 6 | | |
| **Field contribution** | | | | | | | | | | | | | | | | | | | | | | |
| S | | | 0.151 | | | 0.146 | | | | | 0.161 | | | | | - | | | | 0.113 | | |
| E | | | 0.395 | | | 0.377 | | | | | - | | | | | 0.330 | | | | 0.294 | | |
| H | | | 0.294 | | | - | | | | | 0.311 | | | | | 0.248 | | | | 0.220 | | |
| D | | | - | | | 0.324 | | | | | 0.368 | | | | | 0.288 | | | | 0.255 | | |
| A | | | 0.160 | | | 0.153 | | | | | 0.160 | | | | | 0.134 | | | | 0.118 | | |

**Table S3** Prediction of peptide toxicity.

| Subject | Sequence | ML Score | MERCI Score (+ve) | MERCI Score (-ve) | Hybrid Score | Prediction | PPV |
| --- | --- | --- | --- | --- | --- | --- | --- |
| D1 | KLAKAALRARRIDWGDLLRL | 0.325 | 0 | -0.5 | 0 | Non-Toxin | 0 |
| D2 | KIAKVALRARRIDRGDLLRL | 0.255 | 0 | -0.5 | 0 | Non-Toxin | 0 |
| D3 | KLAKAALRARRIDWGDFFRL | 0.345 | 0 | -0.5 | 0 | Non-Toxin | 0 |
| D4 | KIAKAAIRARRIDWGDFFRL | 0.365 | 0 | -0.5 | 0 | Non-Toxin | 0 |
| D5 | KLAKAALRARKIDHGDLLRL | 0.365 | 0 | -0.5 | 0 | Non-Toxin | 0 |
| D6 | KLFRAALRARKIDWGDLLRL | 0.395 | 0 | -0.5 | 0 | Non-Toxin | 0 |

**Table S4** The transmembrane regions prediction of peptides.

| **Peptides** | **The position of the transmembrane region in the sequence** | **CellPPD** |
| --- | --- | --- |
| **D1** | **no** | **CPP** |
| **D2** | **no** | **CPP** |
| **D3** | **no** | **CPP** |
| **D4** | **no** | **CPP** |
| **D5** | **no** | **CPP** |
| **D6** | **no** | **CPP** |
